# *Eggerthella lenta* DSM 2243 Alleviates Bile Acid Stress Response in *Clostridium ramosum* and *Anaerostipes caccae* by Transformation of Bile Acids

**DOI:** 10.3390/microorganisms10102025

**Published:** 2022-10-13

**Authors:** Kristian Jensen Pedersen, Sven-Bastiaan Haange, Kateřina Žížalová, Alina Viehof, Thomas Clavel, Martin Leniček, Beatrice Engelmann, Lukas Y. Wick, Frank G. Schaap, Nico Jehmlich, Ulrike Rolle-Kampczyk, Martin von Bergen

**Affiliations:** 1 Helmholtz-Centre for Environmental Research—UFZ GmbH, Department of Molecular Systems Biology, 04318 Leipzig, Germany; 2 Institute of Medical Biochemistry and Laboratory Diagnostics, First Faculty of Medicine and General University Hospital in Prague, Charles University, Kateřinská 32, 12108 Prague, Czech Republic; 3 Functional Microbiome Research Group, Institute of Medical Microbiology, RWTH University Hospital, 52074 Aachen, Germany; 4 Helmholtz-Centre for Environmental Research—UFZ GmbH, Department of Environmental Microbiology, 04318 Leipzig, Germany; 5 Department of Surgery, NUTRIM School of Nutrition and Translational Research in Metabolism, Maastricht University, 6229 Maastricht, The Netherlands; 6 Department of General, Visceral and Transplant Surgery, University Hospital Aachen, 52074 Aachen, Germany; 7 German Centre for Integrative Biodiversity Research (iDiv) Halle-Jena-Leipzig, Puschstraße 4, 04103 Leipzig, Germany; 8 Institute of Biochemistry, Faculty of Biosciences, Pharmacy and Psychology, University of Leipzig, 04103 Leipzig, Germany

**Keywords:** gut microbiome interaction, bile acids, eggerthella lenta, hydroysteroid dehydrogenase, metaproteomics, metabolomics

## Abstract

Bile acids are crucial for the uptake of dietary lipids and can shape the gut-microbiome composition. This latter function is associated with the toxicity of bile acids and can be modulated by bile acid modifying bacteria such as *Eggerthella lenta*, but the molecular details of the interaction of bacteria depending on bile acid modifications are not well understood. In order to unravel the molecular response to bile acids and their metabolites, we cultivated eight strains from a human intestinal microbiome model alone and in co-culture with *Eggerthella lenta* in the presence of cholic acid (CA) and deoxycholic acid (DCA). We observed growth inhibition of particularly gram-positive strains such as *Clostridium ramosum* and the gram-variable Anaerostipes cacae by CA and DCA stress. *C. ramosum* was alleviated through co-culturing with *Eggerthella lenta*. We approached effects on the membrane by zeta potential and genotoxic and metabolic effects by (meta)proteomic and metabolomic analyses. Co-culturing with *Eggerthella lenta* decreased both CA and DCA by the formation of oxidized and epimerized bile acids. *Eggerthella lenta* also produces microbial bile salt conjugates in a co-cultured species-specific manner. This study highlights how the interaction with other bacteria can influence the functionality of bacteria.

## 1. Introduction

Over the past decades, it has become increasingly evident how crucial the gut microbiota is in health and disease. The gut microbiome protects from pathogens [1] and plays a major role in the synthesis of vitamins [2], as well as in the digestion of complex food components [3]. The metabolites from these processes function not only as nutrients, but also as signaling molecules that can mediate the communication between gut and host. Bile acids are of particular interest in cross-communication due to their widespread systemic effects [4,5,6,7] and their capabilities to directly modulate gut-microbiome composition [8,9,10]. Their systemic effects are due to their ability to bind to nuclear receptors such as Farnesoid X receptor (FXR) and transmembrane receptors such a G protein-coupled bile acid receptor 1(TGR-5) [4,11,12]. The direct effect of bile acids shaping the microbiome can, for instance, be seen in the development of the microbiome in infant animals [13] as well as in defining the response of the microbiome of patients to the alterations in the gut topology after bariatric surgery [14]. It was also shown in mice that if bile flow is obstructed, it resulted in bacterial overgrowth and mucosal injury in the small intestine, which could be reverted in mice still expressing the FXR receptor, but not in ones lacking [5]. Bile acids are amphiphilic molecules and their toxicity towards bacteria is related to their hydrophobicity, and it is known that increasing hydrophobicity leads to increased toxicity [15,16,17,18]. Bile acids are synthesized in the liver from cholesterol via two biochemical pathways, which in humans result in cholic acid (CA) and chenodeoxycholic acid (CDCA), collectively termed primary bile acids [19]. In the liver, they are conjugated with either glycine or taurine and stored in the gallbladder until they are released into the duodenum upon stimulation [20]. In the small intestine, they facilitate the solubilization of lipids and lipid-soluble vitamins, and as they travel through the small intestine approximately 95% are actively reabsorbed across the distal small intestinal wall, in a process termed enterohepatic circulation, which functions as a negative feedback loop that regulates their own biosynthesis [21]. The remaining 5 % enter the large intestine where they primarily undergo extensive chemical modifications by bacteria [22]. Once primary bile acids have been modified by bacteria they are referred to as secondary bile acids, where 7α-dehydroxylation of CA and CDCA results in the more hydrophobic deoxycholic acid (DCA) and lithocholic acid (LCA) respectively. Physiological concentrations and compositions vary greatly through the body and also depend on external factors such as diet [23]. Generally, total bile acid concentration in the colon is between 200–1000 µM where DCA is estimated to be between 10–200 µM [24,25,26]. Some of the chemical modification that bile acids will undergo, whilst passing through the gastro-intestinal tract are deconjugation, dehydroxylation, oxidation, epimerization and, most recently, reconjugation [26,27]. The most widespread bile acid modification is the ability to deconjugate bile acids from the amino acid moieties, via microbial bile salt hydrolases (BSH) [28]. Dehydroxylation is a multistep pathway executed by enzymes that are encoded in the bile acid inducible (bai) operon, which is not common in commensal bacteria [29]. Strains that possess the bai operon are members of the Clostridium cluster XIVa [30,31]. Another widespread enzymatic function is dehydrogenation (oxidation) via hydroxysteroid dehydrogenases (HSDHs). HSDHs catalyze oxidation of the hydroxyl groups at the C-3, C-7 and C-12 position of the steroid core of bile acids [32,33,34]. Oxidation of the hydroxyl groups is stereospecific, since there are both α and β HSDHs. Hence a sequential oxidation and reduction of an OH group can result in epimerization at a given position [35]. Oxidation of the hydroxyl groups is of particular importance due to both the disruption of the amphiphilic structure of bile acids, but also since it hinders the possibility for dehydroxylation to occur [31]. *Eggerthella lenta* (*E. lenta*) (DSM 2243) is known to be a potent bile acid modifier expressing a 3, 7 and 12α HSDH [25,32], making it an interesting strain to co-cultivate with, to understand the significance of how these bile acid forms influence microbe–microbe interactions. It has recently been discovered that the gut microbiome is also capable of performing reconjugations of bile acids, forming bonds with amino acids other than glycine and taurine, which was a previously unknown mechanism. Initially, phenylalanocholic acid, tyrosocholic acid and leucocholic acid were discovered, with more than 50 different bile acid modifications currently identified in cultures of human fecal bacteria [26,36,37,38]. Structural differences in the cell envelopes of gram-negative and gram-positive strains influence how sensitive the bacteria are towards bile acids. Due to the differences in charge as well as gram-negatives possessing two cellular membranes, gram-positive bacteria are more sensitive towards bile acids [21,39,40]. Hence, with the number of secondary bile acids increasing and novel bile acid modifying mechanisms being discovered, it is of growing importance to know specifically which strains are capable of performing such modifications, and also what the consequence of such modifications are both within and between bacteria. In this study, we aim to investigate what the molecular responses to bile acid stress are, and what the significance is of being co-cultivated with a known bile acid modifier using metabolomics and proteomics. In order to gain such insights, we cultivated strains from the simplified intestinal human microbiota extended (SIHUMIx) [41] and co-cultured the individual strains with *E. lenta*.

## 2. Materials and Methods

### 2.1. Bacterial Strains

The bacterial strains chosen for this study are from SIHUMIx, a model system of for the human intestinal microbiota [41], consisting of eight bacterial species *Anaerostipes caccae* (DSMZ 14662), *Bacteroides thetaiotaomicron* (DSMZ 2079), *Bifidobacterium longum* (NCC 2705), *Blautia producta* (DSMZ 2950), *Clostridium butyricum* (DSMZ 10702), *Clostridium ramosum* (DSMZ 1402), *Escherichia coli K-12* (MG1655), *Lactobacillus plantarum* (DSMZ 20174). The strain used for co-culturing was *Eggerthella lenta* (DSMZ 2243). Strains were cultivated as single strains in Yeast-Hemin-Brain-Heart-Infusion (BHI) medium under anaerobic conditions at 37 °C and 175 rpm shaking, maximum 72 h before bile acid stress assays were conducted. See Table S1 for BHI composition. 

### 2.2. Bile Acid Stress Assay

At a maximum of 72 h before the assay(s) were conducted, strains were thawed individually in BHI media as described above. Shortly before inoculation, the type of anaerobic culture tubes (hungates) were supplemented with 600 µM CA (Sodium cholate hydrate, Sigma Aldrich, St. Louis, MO, USA) dissolved in H_2_O or 200 µM DCA (Deoxycholic acid, Merck, Kenilworth, NJ, USA) dissolved in 70% EtOH and gassed with pure N_2_. Strains were inoculated into the hungates in replicates of 6, with a start OD600 = 0.15 if grown as a single culture or 0.075 of each strain when co-cultured. Cultures were measured hourly on a Nanocolor^®^ UV/VIS II, Macherey Nagel at 600 nm. After 6 h of growth, 2 mL bacteria cell suspension was centrifuged (3200× *g*; 10 min; 4 °C) supernatant and pellet were separated and both immediately frozen at −80 °C. 

### 2.3. Bile Acid Measurements

For the bile acid determination, the Bile acid Kit from Biocrates (Innsbruck, Austria) was used. The exact description of the procedure can be found in [42]. All solvents and chemicals were of UPLC/MS grade. 10 µL of standards and internal standards mixture were pipetted onto the filter spots suspended in the wells of the 96-well filter plate. The filter plate was fixed on the top of a deep-well serving as a receiving plate for the extract (a combi-plate structure). In addition, quality controls were distributed on the plate. On the one hand, one QC each was measured with low, medium and high concentrations of the calibration range and additionally three further medium QC samples were distributed over the plate. A 7-point calibration curve (concentration range: 10 to 75,000 nmol/L bile acid specific) was used for calibration. Samples of 10 µL were pipetted on the spots of the kit plate, followed by nitrogen drying. Then, 100 µL methanol was added to the wells, and the combi-plate was shaken for 20 min. The combi-plate was centrifuged to elute the methanol extract into the lower receiving deep-well plate, which was then detached from the upper filter plate. After adding 60 µL Milli-Q water to the extracts, the samples were analyzed. 5 µL of each sample were injected using Acquity UPLC System (Waters) (UHPLC Column from Biocrates P.-No 91220052120868). Initial solvent was 65% A (solvent A: 100% H_2_O, containing 10 mmol/L ammonium acetate), solvent B: 100% acetonitrile containing 10 mmol/L ammonium acetate and 0.1% formic acid (FA) at a flow rate of 0.5 mL/min at 50 °C. Gradient elution was performed using a Q-Trap 5500 mass spectrometer (Sciex). Mass spectrometric detection was employed with electrospray ionization in negative ion mode (IS—4500 eV). Individual bile salts were monitored in MRM windows. Data was processed using Analyst 1.7.1. All peaks were visually inspected for correct integration including the internal standards using the quantification software from Analyst. AUC values were corrected for recovery of the assigned internal standard. Quadratic regression was used to calculate concentrations from standard curves. Afterwards, a further quality control was carried out using the Met IDQ software (version Oxygen) provided by the Biocrates company.

### 2.4. Zeta Potential Assay and Measurements

Strains were inoculated into hungates containing 10 mL BHI (pH = 7.01) in replicates of 3, with a start OD_600_ = 0.1 either with or without the bile acids already in the media. Once strains reached an OD600 = 1 they were centrifuged (970 g, 20 °C, 10 min) in the hungates, hereafter the supernatant was removed. The remaining pellet was washed with 10 mM KNO_3_ (pH = 7), centrifuged as described before and washed again. After washing, pellets were resuspended in 500 µL of 10 mM KNO_3_ and from this solution 50 µL were transferred into 10 mL of 10 mM KNO_3_ and vortexed well. From this, 1 mL was used to fill up a capillary cell (DTS1070, Malvern Panalytical Ltd.) and measured at room temperature on the Zetasizer Ultra (Malvern Panalytical Ltd.) equipped with a He-Ne (633 nm) laser. The obtained data were analyzed with the ZS XPLORER (version 2.3.1.4) and then extracted into GraphPad Prism (version 9.4.0 for Windows, GraphPad Software, San Diego, California USA, www.graphpad.com.) Cell-free BHI controls supplemented with CA or DCA were also measured, but here ZP was = 0 (See Supplementary data). Cell cultures grown without bile acids were measured and used to calculate the ∆ZP to either CA or DCA cultures. 

### 2.5. Preparation of Proteomic Samples

Subsequently, 2 mL bacterial pellets were harvested at the end of the 6 h bile acid stress assay and resuspended in CH_3_OH/H_2_O/CHCL_3_ (2:1:1, v:v:v). Following resuspension, samples were incubated on ice for 10 min and were then sonicated and centrifuged (1700× *g*; 10 min; 4 °C). Samples were then dried in a speed vac and resuspended in UT buffer (8 M Urea; 2 M thiourea). Protein concentration was determined on a NanoDrop 2000c (Thermo Scientific, Rockford, IL, USA) 4 µg of protein in UT-solution was filled with up to 20 µL with 20 mM ammonium bicarbonate followed by addition of 2 µL of 25 mM dithiothreitol solution for disulfide reduction and the samples were incubated via Thermoshaker (60 °C; 1 h; 1400 rpm). This was followed by alkylation, by adding 14 µL of 20 mM ammonium bicarbonate and 4 µL of 100 mM 2-iodoacetamide and incubation at 37 °C for 30 min at 1400 rpm. Overnight enzymatic digestion was performed with trypsin (Promega, Madison, WI, USA) at 37 °C. Extracted peptides were purified by SOLAµ (Thermo Scientific, Waltham, MA, USA) as per the manufacturer’s recommendation. After evaporation peptides were resuspended in 20 µL 0.1% FA.

### 2.6. Proteomic Measurements

For each LC−MS run, 1 μg of peptides was injected into a Nano-HPLC (UltiMate3000, Dionex, Thermo Fisher Scientific, Waltham, MA, USA). Peptides were trapped for 3 min on a C18-reverse phase trapping column (Acclaim PepMap 100, 75 μm × 2 cm, particle size 3 μM, nanoViper, Thermo Fisher Scientific), followed by separation on a C_18_-reverse phase analytical column (Acclaim PepMap100, 75 μm × 25 cm, particle size 3 μM, nanoViper, Thermo Fisher Scientific). A two-step gradient was used with A: 0.01% FA in H_2_O and B: 80% ACN in H_2_O and 0.01% FA in mobile phases. The first step of the gradient was 90 min, where B went from 4% to 30%, then 30 min where B went from 30% to 55%. This was followed by 30 min where B went from 55% back to 4%, with a flow rate of 300 nL/min and a column temperature of 35 °C. The eluted peptides were ionized by a nano ion source (Advion Triversa Nanomate, Ithaca, NY, USA) and detected via a Q Exactive HF-MS (Thermo Fisher Scientific) with the following settings: Scan range 150–2000 m/z, MS resolution 120,000, MS automatic gain control (AGC) target 3,000,000 ions, maximum injection time for MS 80 ms, intensity threshold for MS/MS of 17,000 ions, dynamic exclusion 30 s, TopN = 20, isolation window 1.6 m/z, MS/MS resolution 15,000, MS/MS AGC target 50,000 ions, maximum injection time for MS/MS 120 ms. Mass spectrometric data processing was performed using Proteome Discoverer (v.2.5, Thermo Fischer Scientific, Waltham, MA, USA) with SequestHT search engine. The search settings were set to trypsin (Full), or Asp-N (Full), max. missed cleavage sites 2, precursor mass tolerance 10 ppm, and fragment mass tolerance 0.05 Da. Carbamidomethylation of cysteines was specified as a fixed modification. False discovery rates (FDR) were determined using Percolator. The searches against SequestHT were undertaken with a constructed proteome database containing the reference proteomes downloaded from Uniprot (www.uniprot.org, accessed on 10 Octorber 2022). Data from the searches were further processed as previously described [14]. Protein intensities were converted to relative abundances by dividing the intensity of the protein with the summed intensities of all proteins from the same species detected in the sample. Protein functions and pathway assignment were undertaken using Ghostkoala webapplication from KEGG [43]. Only pathways containing minimum five proteins and a minimum total coverage of 10% were selected for further analysis.

### 2.7. Untargeted Metabolomics Measurement

Prior to analysis, supernatants from the bile acid stress assay were mixed with five volumes of MeOH:ACN:H_2_O in a 2:3:1 (v:v:v) ratio, sonicated for 5 min and centrifuged. 550 µL supernatant was then transferred and evaporated, before being resuspended in 100 µL 0.1% FA and 1% ACN in water. Samples measured in positive and negative ionization mode were further diluted 1:20 and 1:10, respectively. For measurement, 2 or 5 µL (positive and negative ionization, resp.) of each extract was injected onto a HPLC system coupled online with a 6546 UHD Accurate-Mass Q-TOF (Agilent Technologies). Metabolites were separated with an Agilent Zorbax Eclipse Plus C18 column (2.1 × 100 mm, 1.8 µm) equipped with a related pre-column (2.1 × 50 mm, 1.8 µm). The autosampler was kept at 5 °C and column oven was set to 45 °C. Separation was achieved using a binary solvent system of A (0.1% FA in water) and B (0.1% FA in ACN). The gradient was as follows: 0–5.5 min: 1% B; 5–20 min: 1–100% B; 20–22 min: 100% B; 22–22.5: 100–1% B; 22.5–25 min: 1% B. Metabolites were eluted at a constant flow rate of 0.3 mL/min. Eluted compounds were measured with the QTOF operated in centroid mode. Full scan data was generated with a scan range of 50–1000 m/z in positive and negative ionization mode. Out of the survey scan, the two most abundant precursor ions with charge state = 1 were subjected to fragmentation. The dynamic exclusion time after two acquired spectra was set to 0.1 min. Obtained spectral data (.d files) were imported into Progenesis QI software (Non-Linear Dynamics). Different ionization modes and microbial strains were analyzed separately. The adduct ions involved [M + H], [M + H − H_2_O] and [M + H + Na] for positive mode and [M − H], [M − H_2_O − H] and [M + FA − H] for negative mode. In a generic workflow, chromatogram were aligned using an automatically chosen reference chromatogram from the dataset. The following software-guided, peak-picking tool resulted in a data matrix, including the retention time, mass-to-charge ratio and corresponding normalized peak area. The subsequent automated database search based on a ChemSpider plug-in was used as identification method with *E. coli* metabolome database, fecal metabolome database and KEGG as resources. After exporting the results regarding compound measurement and putative identifications for all measured compounds, data was processed, as previously described [14].

### 2.8. Measurements of Keto and Iso Forms of Bile Acids/MSBCs

Bile acids and their oxidized forms were measured as previously described [44]. The data is semiquantitative since there were no standards available for all the different modifications at the time. Hence, the data is given as a percentage compared to the peak area of the parent bile acid. Furthermore, this lack of standards also refrains from distinguishing between the different keto and iso forms; hence, CA isoforms will be referred to as isoCA 1–3, and the single keto forms as monoketoCA 1–3. Likewise, keto forms of DCA will be referred to as monoketoDCA 1–3. For measurement of microbial bile salt conjugates (MBSC)s, 50 μL of the medium was mixed with 10 μL of internal standard (d4GCDCA), deproteinized by three volumes of 2-propanol/acetonitrile (1:2), vortexed, and centrifuged at 13,000 g for 15 min at room temperature. The supernatant was dried at 65 °C under nitrogen, dissolved in 50 μL of 50% methanol and analyzed. Gradient for MBSC assay was slightly modified (methanol concentrations were as follows: 0–1 min 50%; 1.0–10.0 min 95 %; 10–14 min 95 %; 14–18 min 50%). Following in-house prepared standards were used: Leu-CDCA, Leu-CA, Phe-CDCA, Phe-CA, L-Tyr-CDCA, D-Tyr-CDCA, L-Tyr-CA, D-Tyr-CA, 3,7-diketo CA, 3,12-diketo DCA, 3,7,12-triketo CA. For qualitative use, standards of 3-ketoBA and 7-ketoBA were prepared from individual BA using either 3α-hydroxysteroid dehydrogenase (Reagent B: Bile acids kit 450-A, Trinity Biotech (Wicklow, Ireland) or 7α-hydroxysteroid dehydrogenase (in-house prepared recombinant HdhA from *E. coli* GC-10). CDCA conjugates (with Leucine, Phenylalanine and Tyrosine) were used to tune mass spectrometer parameters for detection of respective DCA derivatives. Media blanks containing either CA or DCA were averaged and subtracted from their respective categories within each strain.

### 2.9. Omics-Data Statistical Analysis

The statistics for proteomics and untargeted metabolomics were as previously described [14]. Principal component analyses (PCA) were conducted using the VEGAN package [45]. For single variables, Student’s *t* test were preformed and where appropriate (number of tests > 20), *p*-values were corrected for multi-testing by the Benjamini–Hochberg method [46], and all other figures were constructed using the R package ggplots2 [47].

## 3. Results

### 3.1. Growth in the Presence of Bile Acids in Single Species Culture and Co-Cultures with E. Lenta

**Figure 1:**
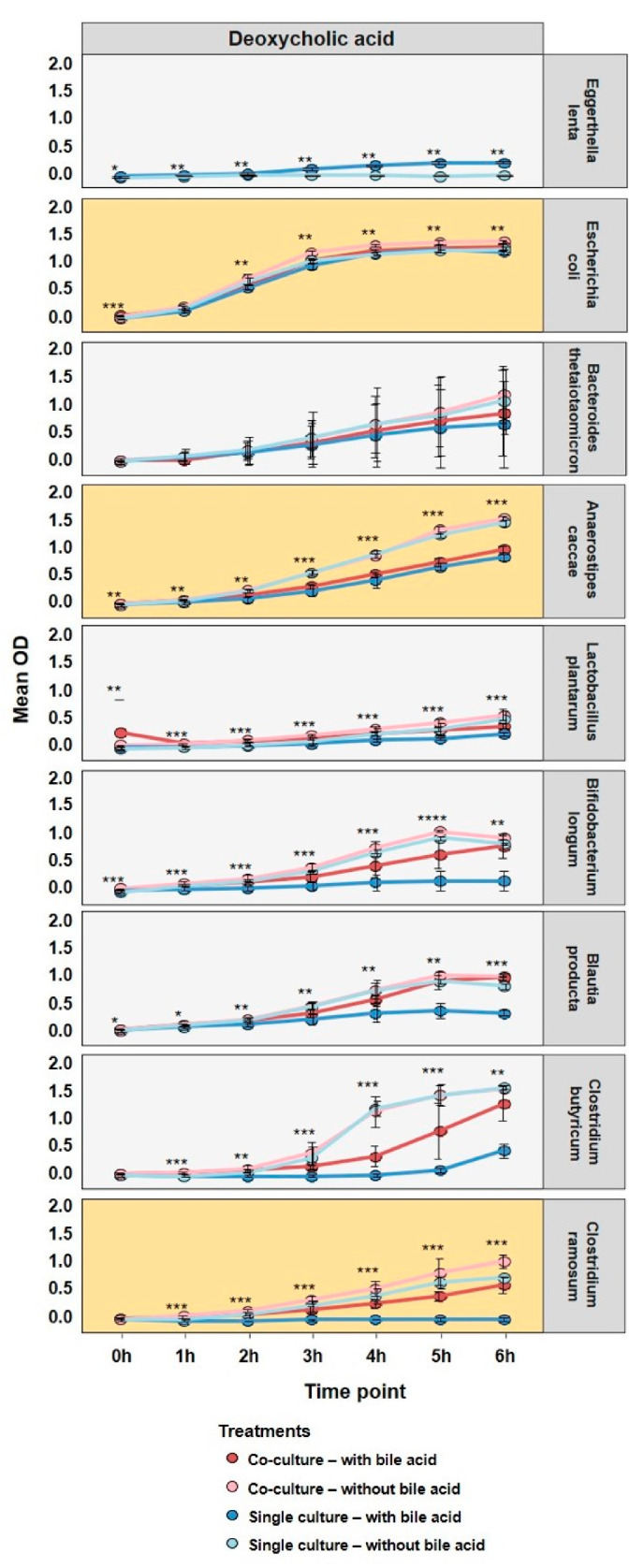
Growth of SIHUMIx species in single and co-culture with *E. lenta* with treatment 200 µM DCA. For each experiment, six replicates were analyzed and the line represents the mean. Strains are ranked according to increasing inhibition of growth when stressed with DCA. Error bars are standard deviations. Statistical significance was determined using ANOVA analysis, * *p* < 0.05, ** *p* < 0.01, *** *p* < 0.005, **** *p* < 0.001.

Growth curves from the bile acid stress experiments are displayed in Figure 1; the ranking is organized according to increased inhibition of growth caused by DCA. Amounts of 600 µM of CA and 200 µM DCA was selected since these are physiologically relevant concentrations encountered in the colon [19,24,48] and previous experiments suggested these concentrations to be fitting (Supplementary Figure S1). For growth curves of CA stress, see Supplementary Figure S2 and for growth data please, see Supplementary data.

When cultures were stressed with CA, there is no inhibition of growth in any strain; in the case of *E. lenta*, it even suggests that it grows slightly better. There are also no clear effects of co-culturing under CA stress, other than a slightly elevated final OD. When considering DCA stress, again *E. lenta* seems to feature an increased growth, whereas *A. caccae*, *B. longum*, *B. producta*, *C. butyricum* and *C. ramosum* are significantly inhibited. In the cases of *B. longum*, *B. producta*, *C. buturicum* and *C. ramosum* co-culturing seems to alleviate stress from DCA. These results suggest that the presence of *E. lenta* alleviates the bile acid toxicity, in particular for gram-positive strains, such as *C. ramosum*. For further analysis, *E. coli*, *A. caccae* and *C. ramosum* were selected as they showed a low, middle and pronounced stress response to DCA and co-culture. Furthermore, bile stress response in bacteria strongly depends on their type of membrane, where *E. coli* is gram- negative, *A. caccae* is gram-variable [49] and *C. ramosum* is gram-positive. 

### 3.2. Concentrations of CA and DCA in Single Species Culture and Co-Cultures with E. lenta

In order to verify the concentrations of CA and DCA at the end of bile acid stress assays, supernatants from these cultures were measured and concentrations determined.

**Figure 2:**
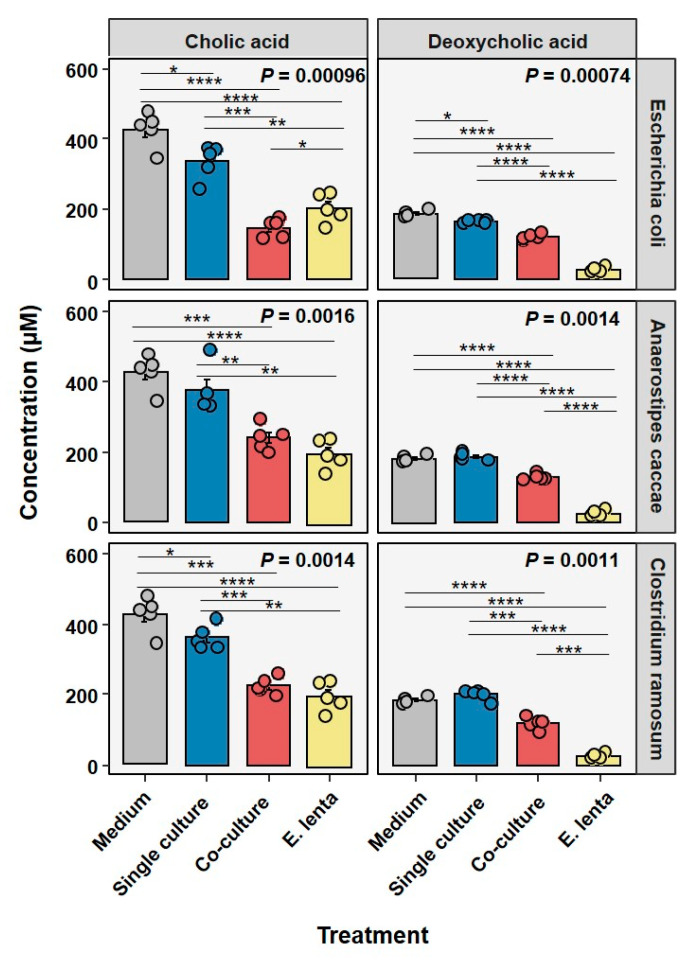
Determination of bile acid concentrations in single and co-culture after 6 h of incubation. For every sample, five replicates were measured and the bars represent the mean value. Group statistics were calculated by ANOVA with post-hoc pairwise Students *t*-test. (* *p* < 0.05, ** *p* < 0.01, *** *p* < 0.001 and **** *p* < 0.0001).

As seen in Figure 2, all single cultures are capable of transforming CA to a slight extent, but this significantly increases when co-cultured with *E. lenta*. On average single cultures of *E. coli* and *C. ramosum* the concentration decreased by 90.8 ± 43.3 µM and 65 ± 30.9 µM respectively, whereas co-cultures decreased concentrations by 281.2 ± 22.8 µM and 202 ± 22.9 µM. In the case of *E. coli,* the co-culture decreases the concentration more than *E. lenta* on its own. In the case of DCA the only single culture that could significantly decrease concentration was *E. coli* that on average decreased it by 20.3 ± 3.8 µM. In all other cases co-culturing significantly, decreased concentrations compared to their respective single culture, with an average of 61.5 ± 7.2 µM for *E. coli* co-cultures, 54.5 ± 7.7 µM for *A. caccae* co-cultures, and 64.7 ± 16.1 µM for *C. ramosum* co-cultures. Interestingly, single cultures of *E. lenta* outcompeted all co-cultures in decreasing DCA concentration, with an average decrease of 157.7 ± 5.4 µM. 

With the indication that bile acid concentrations decrease in particular in the presence of *E. lenta*, and the alleviation of growth inhibition, we investigated the membrane integrity. The remaining bile acids covered in the biocrates kit, can be seen in Supplementary Figure S3.

### 3.3. Assessment of Membrane Integrity by Zeta-Potential of E. coli, A. caccae, and C. ramosum in Single Species Culture and Co-Cultures with E. lenta

Zeta potential (ZP) measurements were performed to approach the effects of bile acids on the membrane. ZP is an electrochemical property that represents the potential at the shear plan of an electrical double layer encompassing a cell in an ionic solution. It provides critical information about cell-surface characteristics [50] and can reflect the physiological status of cells after, e.g., exposure to chemicals and nanoparticles [51,52]. Hence, ZP is being more frequently used to approach cell membrane integrity [53,54]. ZP values can be seen in Supplementary data and examples of full chromatograms of the ZP measurements can be seen in Supplementary Figure S4. 

**Figure 3:**
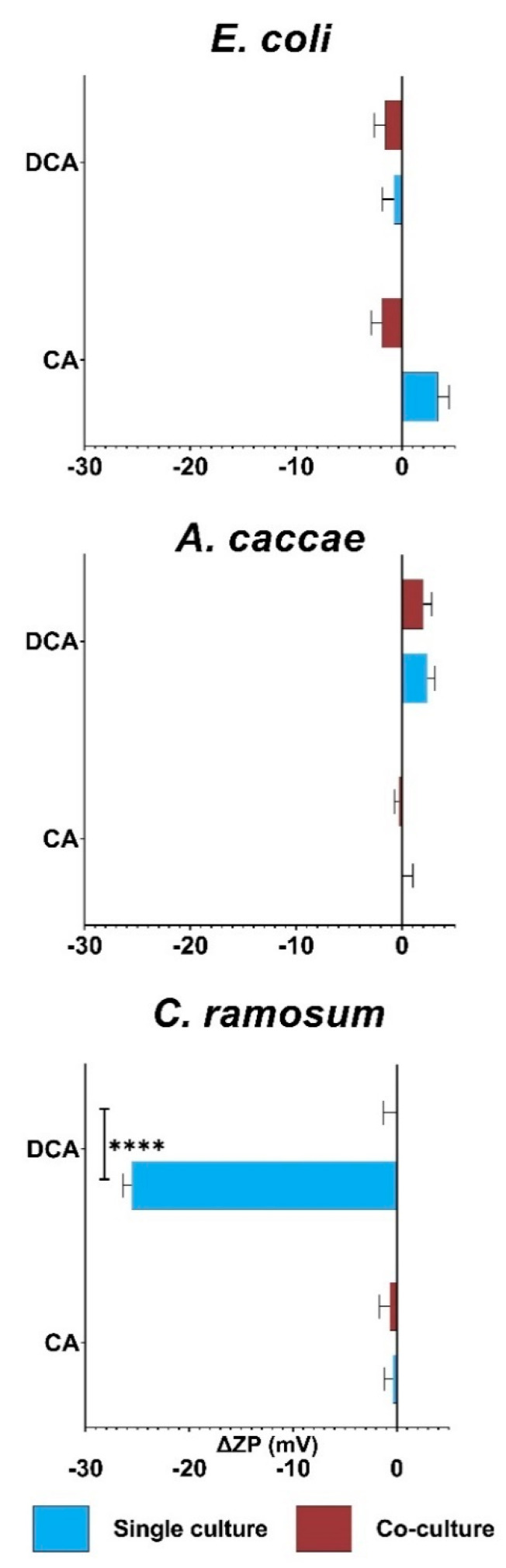
Relative change in zeta potential of cells in log phase exposed to bile acids. The change in ZP is compared to their respective blanks and is shown in mV. For all analyses, three replicates were analyzed and bars represent the mean value. Samples were harvested when OD_600_ = 1.0 Errors bars are standard deviations. Statistical significance was determined using a Student’s *t*-test, **** *p* < 0.001.

The change in ZP compared to their respective blanks of bacterial cultures stressed with CA or DCA can be seen in Figure 3. In this study, the average ZP for untreated cultures were found to be −51 (SD 0.81 +/−) and −54 (SD 01.14 +/−) mV for *E. coli* single and co-culture, −32 (SD 0.93 +/−) and −31 (SD 0.04 +/−) mV for *A. caccae* single and co-culture and −19 (SD 0.73 +/−) and −20 (SD 1.27 +/−) for *C. ramosum* single and co-culture (See Supplementary data). It can be observed that the gram-negative strain *E. coli* has the most negative ZP, the ZP of *A. caccae* the gram-variable is slightly less negative than *E. coli* and that the gram-positive strain *C. ramosum* has the least negative ZP. This is expected due to gram-negative bacteria possess the negatively charged LPS layer. As evident, neither bile acid nor co-culturing have any significant effect on either *E. coli* or *A. caccae*. When the single culture of *C. ramosum* is observed, it suggests that CA does not affect the ZP, whereas DCA on average decreases the ZP by −26 mV. Co-culturing reverts this decrease of ZP seen from single cultures to levels comparable with the blank. 

Hence, *A. caccae* and *E. coli* show no significant changes in ZP from either bile acid, whereas *C. ramosum* again shows a clear protective effect when co-cultured. These results support the finding from the growth experiments that the presence of *E. lenta* alleviates bile acid toxicity for gram-positive bacteria in particular, and that *E. coli* and *A. caccae* seems unaffected on their membrane. This led us to conduct deeper functional investigations of the selected strains.

### 3.4. Proteomics Reveals Specific Responses to DCA Depending on Membrane Charecteristics and the Presence of E. lenta

**Figure 4:**
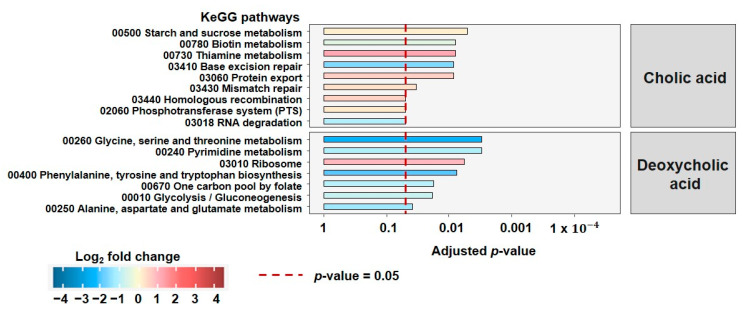
Molecular response of *C. ramosum* towards CA and DCA in co-culture with *E. lenta* vs. single culture. Depicted are the significantly altered KEGG metabolic pathways based on the relative abundance of proteins detected. The proteins stem from the bacterial pellet harvested from the end of the bile acid stress assays. The direction of the change is color-coded, red indicates an increase, blue a decrease in the fold change in the co-culture.

**Figure 5:**
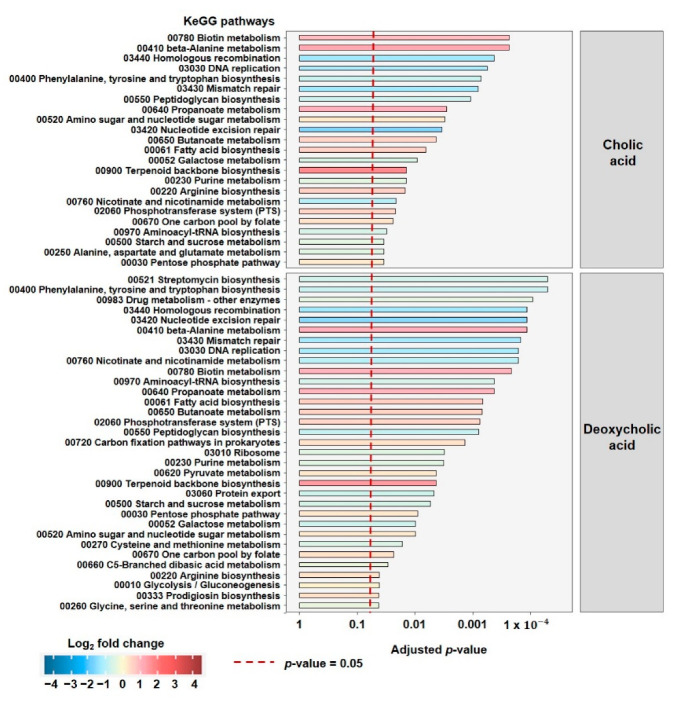
Molecular response of *A. caccae* towards CA and DCA in co-culture with *E. lenta* vs. single culture. Depicted are the significantly altered KEGG metabolic pathways based on the relative abundance of proteins detected. The proteins stem from the bacterial pellet harvested form the end of the bile acid stress assays. The direction of the change is color-coded, red indicates an increase, blue a decrease in the fold change in the co-culture.

The growth curves indicated that gram-positive strains are more susceptible to the effects of DCA than gram-negative species. Secondly, this growth inhibition can be alleviated in the presence of *E. lenta*. With respect to growth, there is only a slight effect detectable in the gram-variable *A. caccae*. After estimating the extent of membrane-associated effects by assessing the ZP, we wondered how the selected strains remodeled their structure and metabolism when stressed with CA and DCA and, therefore, analyzed the effects of co-culturing by (meta)proteomics (Figure 4 and Figure 5). Pathways depicted on Figure 4 and Figure 5 represent fold changes within the represented bacterium, where the direction of the change is color-coded, with red indicating an increase, blue indicating a decrease in the fold change in the co-culture. Pellets from the end of the bile acid stress assays were analyzed. The proteomic analysis yielded only very few, significantly altered, affected pathways in *E. coli* due to co-culturing, consistent with no effect on either growth or ZP. For *C. ramosum*, we detected 16 significantly altered pathways (Figure 4) and 55 for *A. caccae* (Figure 5). Only pathways containing a minimum of five proteins and a minimum total coverage of 10% were selected for further analysis. For relative species abundance and PCAs of proteomic data, please see Supplementary Figures S5 and S6. 

Although there is a clear increase in growth in the presence of *E. lenta*, only a limited number of pathways are changed significantly in *C. ramosum*. For DCA the pathways of “glycine, serine and threonine metabolism”, “phenylalanine, tyrosine and tryptophan metabolism”, and “alanine, aspartate and glutamate metabolism” are downregulated in the co-culture. The same is true for the pathway of “Glycolysis” being down-regulated in the co-culture under DCA stress. The increased growth of *C. ramosum* in co-culture is reflected in the increase in the “ribosome pathway”.

For CA, the decrease in the pathways “Base excision repair” and “RNA degradation” points to the alleviation of the genotoxic effects of bile acids in co-culture. The effects on the other pathways, except for induction of “thiamine metabolism” were not overly strong, and this is in line with the non-significant changes with respect to growth. 

Interestingly, we found more affected pathways *A. caccae* (Figure 5), which showed a DCA-dependent but not a co-culture dependent effect in growth.

For both DCA and CA, we found a strong decrease for pathways such as “mismatch repair”, “homologous recombination” and “nucleotide excision repair” that are linked to genotoxic effects that might be alleviated in the presence of *E. lenta*. 

Even though we see only slight effects on growth, many pathways were elevated that are linked to increased growth such as carbon fixation, propanoate metabolism, butanoate metabolism, and fatty acid biosynthesis. In DCA, more pathways related to the synthesis of amino acids are affected. These results indicate that mechanisms related to growth are increased under these conditions, in combination with a decreased need to produce amino acids. The effect of co-culture, in the presence of both CA and DCA is especially strong on upregulated “Terpenoid backbone synthesis”.

### 3.5. Metabolomics Reveals Metabolites Related to Altered Pathways Detected in Proteomics

**Figure 6:**
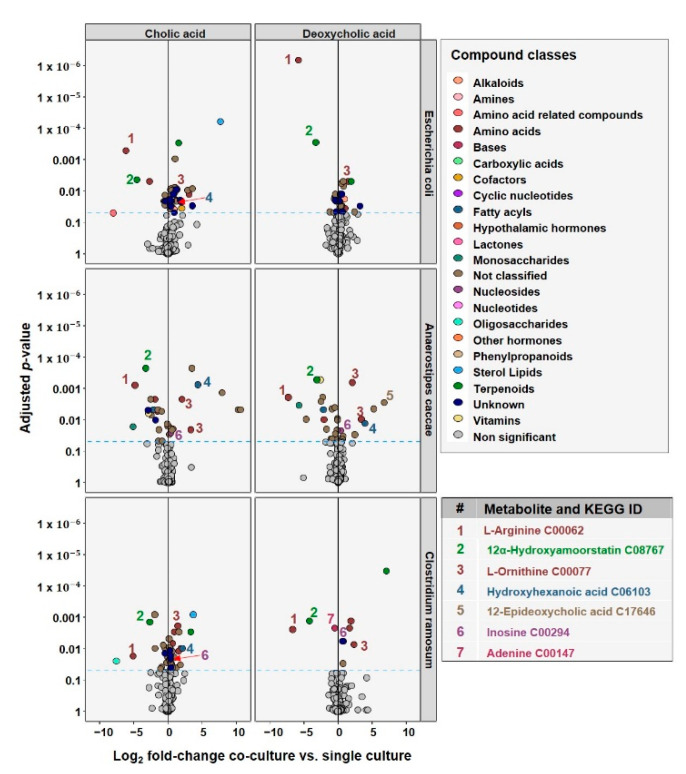
Change in relative abundance of metabolites detected in the medium as calculated by co-culture vs. single culture. Volcano Plot of metabolite Log_2_ fold-change in metabolite abundances plotted against Benjamini–Hochberg adjusted *p*-values. Measurements are from the medium supernatants of cultures grown in the bile acid stress assays. Significant metabolites are color coded by compound class. Dashed blue line indicates *p* = 0.05.

The proteomic response to bile acid stress was validated by untargeted metabolic analyses of the supernatant from the end of the bile acid stress assays, in order to detect metabolites that potentially play a role in the interaction between the co-cultured species. The effects of CA and DCA in single and co-culture with *E. lenta* are depicted in Figure 6. For PCAs of these measurements, please see Supplementary Figure S7.

Similar to the proteomic results, the changes are least prominent in *E. coli*, more abundant in *C. ramosum* and strongest in *A. caccae*. Thus, we focus here on the effects in *C. ramosum* and *A. caccae*. A common feature for all co-cultures is the decrease of a metabolite, which might be L-Arginine (metabolite no 1). This could be related to an increased uptake by *E. lenta*, since it was reported that arginine leads to increased growth in *E. lenta* [55]. Another consistently decreased metabolite in co-cultures is metabolite no. 2, which we could putatively identify as 12α-Hydroxyamoorstatin, which is a rare metabolite but linked to the bile acid structure and thus is potentially indicating to an increased transformation of bile acids by *E. lenta*. In contrast, there is also one metabolite that is consistently increased in co-culture, which could be identified as L-ornithine (metabolite no. 3). L-Ornithine is involved in the synthesis of arginine and an increased uptake of arginine might result in a decrease of ornithine produced and secreted by *E. lenta*. Interestingly, we detected metabolites significantly altered in abundance between the co-culturing with *E. lenta* and single culturing for *A. caccae* and *C. ramosum,* which might be nucleosides. In both mentioned species, for DCA and for CA, we observed a significant increase in the co-culture of metabolite no. 6, which was putatively identify as inosine. Inosine is a degradation product of adenosine. We also observed a decrease in metabolite no. 7, which we putatively identified as adenine, a further degradation product of adenosine, in the co-culture of *C. ramosum* with DCA. 

In *A. caccae* metabolite no. 4, which was suggested to be 6-hydroxyhexanoic acid, was consistently elevated in co-cultures both in the presence of CA and DCA. 6-hydroxyhexanoic acid is part of the bacterial degradation pathway of bile acids [56]. Furthermore, metabolite no. 5, which is suspected to be 12-epideoxycholic acid, was seen to be elevated in *A. caccae* during co-culturing and DCA stress. 12-epideoxycholic acid is an epimer of the C-12 OH group of DCA.

### 3.6. Detection of Oxidized and Eperimized Bile Acids in Single Culture vs. Co-Culture

**Figure 7:**
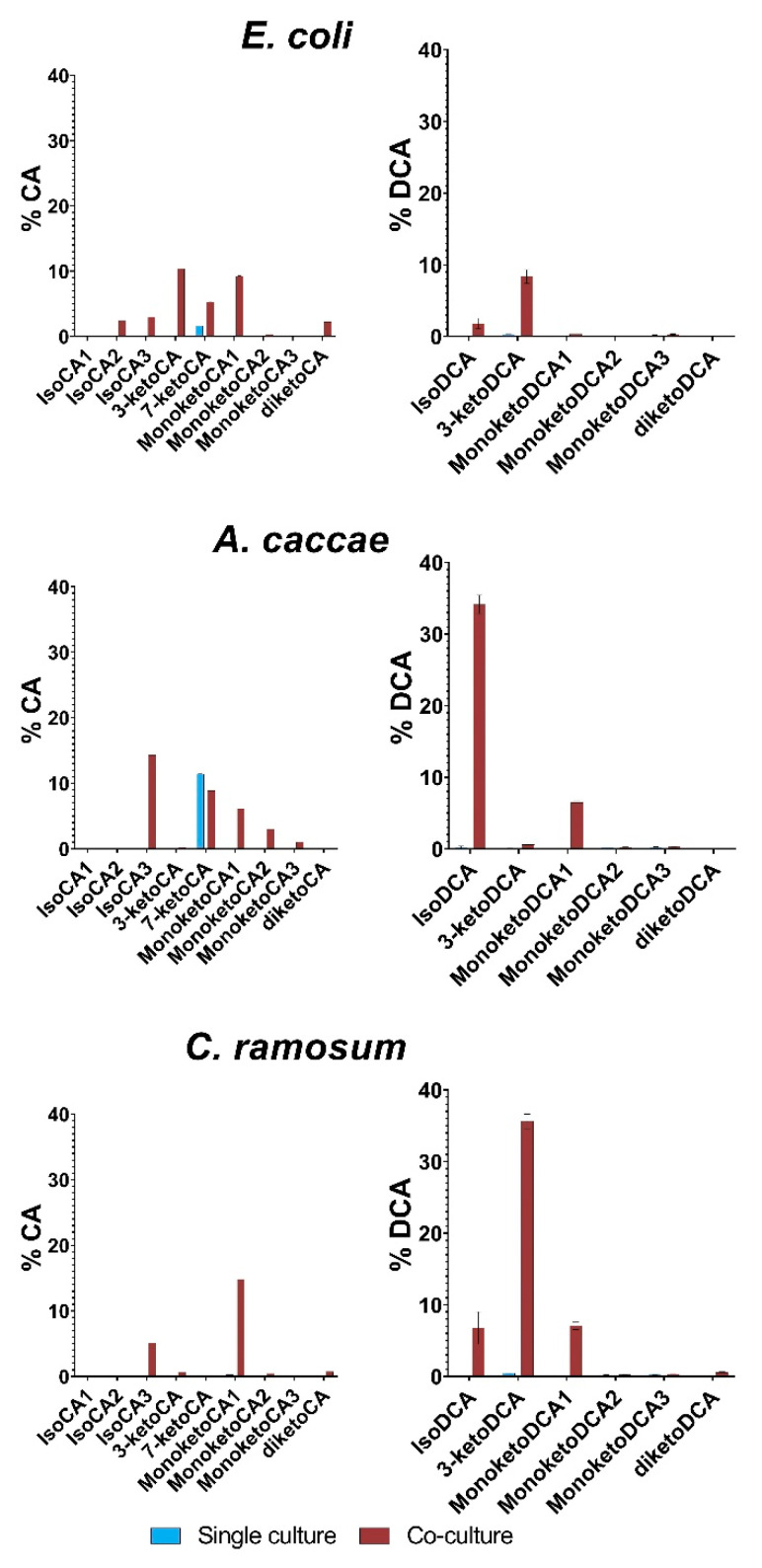
Epimerization and oxidization of CA and DCA in single and co-cultures. The bars represent the mean of three biological replicates and the abundances are ratios compared to the peaks of the original added bile acids due to lack of standard for the different iso and keto forms. Blue is single culture and red is co-culture.

Based on the finding of elevated levels of 12-epideoxycholic in the supernatant of co-culture and the extended repertoire of bile acid modifying enzymes in *E. lenta* [25,32], we tested more comprehensively for bile acid oxidation and epimerization. Figure 7 shows semiquantitative abundances of the iso- (i.e., 3β epimers) and oxo (i.e., keto) forms of CA and DCA from the bile acid stress experiments depicted in Figure 1. The data is semiquantitative since there were no standards available at the time for all the different modifications; hence, the data is given as a%, compared to the peak of its original bile acid. For our definition of nomenclature, see Method section “2.8 Measurements of oxo and iso forms of bile acids/MSBCs”. Keto/iso figure for all 8 SIHUMIx strains can be seen in Supplementary Figure S8.

When single cultures of *E. coli* are exposed to CA stress, only one modification is observed, namely 7-ketoCA. This is line with the fact that *E. coli* expresses a 7α-HSDH (WP_000483353.1, [57]). The corresponding co-culture shows a different pattern with signals for isoCA-2 and 3 as well as monoketoCA 1. 3-ketoCA and 7-ketoCA are also observed. Likewise, *A. caccae* single culture also only shows formation of 7-ketoCA, and indeed a 7α-HSDH homologue (WP_054335312.1, E value = 3 × 10^−37^, identity 32.74%) was found by homology searching using NCBI’s blastp tool against the 7α-HSDH described previously in *E. coli* (See Supplementary Table S2 for HSDH enzyme accession numbers). Co-culturing again results in formation of isoCA 3 and monoketoCA 1–3. Single cultures of *C. ramosum* do not seem to form any iso or keto form of CA. Co-cultures have a different pattern in this case, with isoCA 3 and monoketoCA 1 being the dominant forms. Cultures in the presence of DCA show a more unambiguous trend, since no single culture produces any epimerized or oxidized form with an abundance exceeding 1%. For *E. coli* and *C. ramosum* co-cultures, 3-ketoDCA is readably observed, whereas *A. caccae* co-cultures seem more prone to form isoDCA. It is tempting to speculate that this iso form corresponds to 12-epideoxycholic acid identified in our metabolomic screen, although it cannot be ruled out that it concerns 3β-DCA. The latter would be in line with higher formation of especially 3-ketoDCA helps to explain the reduced toxicity as it potentially reaches 35% of DCA converted. This finding is accordance with the expression of 3α, 3β, 7α, and 12α HSDHs by *E. lenta* [25,32,58]. A novel 12β HSDH was also recently identified, completing the epi-bile acid pathway [59] where homology searching using blastp for this enzyme (WP_027099077.1) against *E. lenta* shows a candidate (WP_114518444.1, E value = 1 × 10^−44^, 36.5% identity). Taken together, these results indicate that the oxidation and epimerization of bile acids is largely dependent on *E. lenta*, and provides a mechanistic framework for reduced bile acid toxicity, in particular, with DCA as a substrate. *E. coli* and *A. caccae* seem to be capable of forming 7-ketoCA on their own, but all other oxidized and epimerized forms are only formed when co-cultured with *E. lenta*. With these results indicating that *E. lenta* seems to be important in the formation of epimerized and oxidized forms of bile acids, we sought to investigate a recently discovered new class of bile acids, namely the MBSCs. 

### 3.7. Detection of Microbial Bile Salt Conjugates in Single Culture vs. Co-Culture

**Figure 8:**
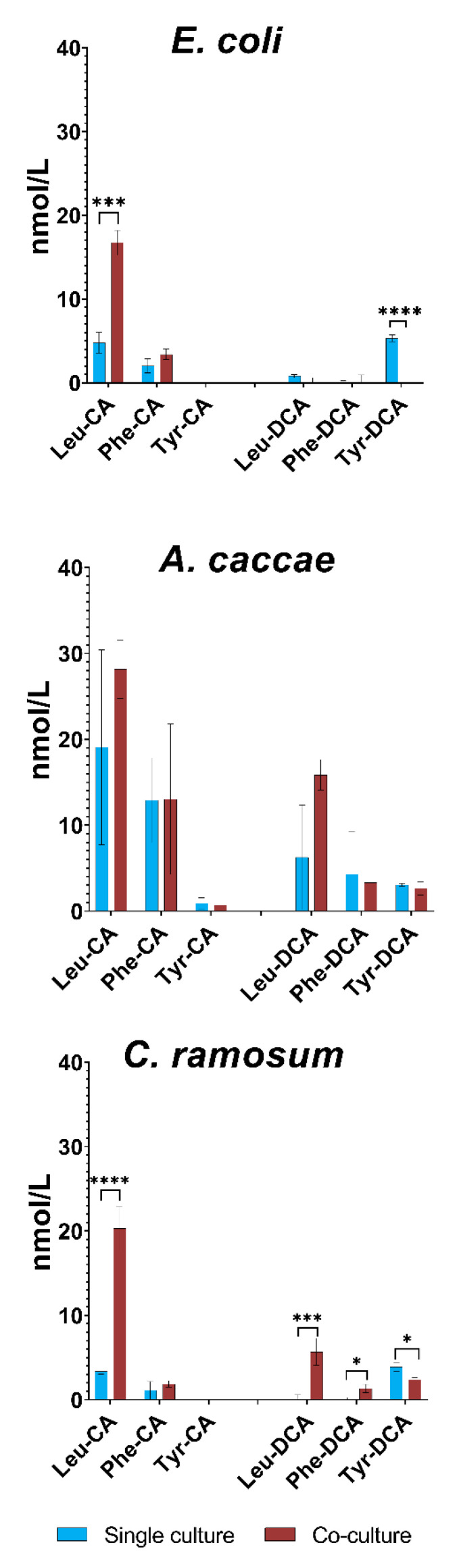
MBSCs produced in single and coculture. Bar charts showing the mean concentration from three biological replicates (nmol/L) of the MBSCs estimated from MS measurements of single and co-cultures. The left hand side of the graph shows the cultures stressed with CA and the right-hand side those treated with DCA. Blue colors are single cultures and red are co-cultures. Error bars are strandard deviation and statistical significance was determined using a two tailed Student’s *t*-test, * *p* < 0.05, *** *p* < 0.005, **** *p* < 0.001.

MBSCs are a novel class of bile acids that are formed by microbial reconjugation of deconjugated/unconjugated bile acids with typically non-canonical amino acids, although host-resembling glycine conjugates can also be generated by microbes [27,36,37]. This novel reaction type was previously not suspected to be in the repertoire of bile acid modifications, but has opened the door to discovering new bile acids. Figure 8 shows the concentrations (nmol/L) of MBSCs from supernatant from the bacterial cultures. Due to the lack of a standard, DCA conjugates are to be considered semi-quantitative since their abundance is based on a *L*-Leu-CDCA standard. MBSC figure for all 8 SIHUMIx strains can be seen on Supplementary Figure S9. Media blanks containing either CA or DCA were averaged and subtracted from their respective categories within each strain.

Comparing *E. coli* single and co-cultures, statistically significant differences are seen for Leu-CA and Tyr-DCA. For Leu-CA, co-culturing increases the concentration, whereas the single culture seems to have a higher abundance of Tyr-DCA compared to co-culture (not detected). *A. caccae* cultures have no significant differences between single and co-culture, so *A. caccae* seems to be capable of producing detectable levels of most of the tested MBSCs by itself. For *C. ramosum*, Leu-CA is again significantly increased in the co-culture and Tyr-DCA is more abundant in the single culture. Leu-DCA and Phe-DCA are also significantly increased in the co-culture since there was no signal in the single cultures. Taken together, these results point towards the capacity of all strains to reconjugate bile acids with Leucine, Phenylalanine and Tyrosine, but that the presence of *E. lenta* in particular elevates levels of Leu-CA. These observations add the studied strains of *E. coli*, *A. caccae, C. ramosum* and *E. lenta* to the range of bacteria capable of non-canonical bile salt conjugation [37].

## 4. Discussion

From the growth curves, it was seen that DCA inhibited growth for *A. caccae* and, in particular, *C. ramosum*, where co-culturing with *E. lenta* could alleviate some of this inhibition for *C. ramosum*, but not for *A. caccae*. This effect was also found to be bile acid specific with a stronger effect of the more hydrophobic DCA compared to CA. Bile acid sensitivity relates to the differences in cellular-envelope composition between gram-negative and gram-positive bacteria. [21]. The slight effect on *A. caccae* was interesting, since nothing was known about the bile acid sensitivity of gram-variable bacteria such as *A. caccae*. This species is described to be gram-positive in the lag phase and becoming more gram-negative during log phase [49]. Hence, it would be interesting to investigate how the proteome of *A. caccae* changes during cultivation, and investigate if there are certain transporters or other mechanisms to deal with bile acid stress. We did not see any response from bile acid stress in *E. coli* and it is also known that gram-negative strains are more bile acid resistant where *E. coli* is known for having mechanisms to cope with bile acid stress [21,60]. In regard to the experimental setup, the harvesting point could have been optimized for the individual stains, since they were all harvested after 6 h. This makes the intre-strain comparison less applicable; for instance, if selected strains are compared, it can be observed that *E. coli* has been in the stationary phase for approximately 4 h, whereas *A. caccae* and *C. ramosum* has just started to reach stationary phase. To gain insights into the specific molecular responses to bile acid stress, earlier time points should have been included as well. An interesting finding was that it seemed that *E. lenta* grew better under bile acid stress conditions. Normally, bacterial growth is inhibited by bile acids, but there are cases where certain strains grow better. *Akkermansia muciniphila* has previously been described to grow better under sodium deoxycholate conditions and actually be more inhibited by oxo-bile acids [61]. Via bile acid measurements, we suggested that co-culturing contributed to a significant decrease in concentration of both CA and DCA across all three strains. This speaks in favor of *E. lenta* being the main driver of bile acid transformations. It should, however, be considered that this decrease could also be caused by an uptake of bile acids into the cells or binding to the cell membranes or envelopes. Our results from ZP measurements indicated that *C. ramosum* had a shift towards a more negative ZP when stressed with DCA. Studies on the effects of antimicrobial peptides on *E. coli* suggest a correlation of ZP shifts towards neutral charge and increased permeability of cell membranes and decreased cell viability resp [53]. This difference could, however, be due to the difference in stressor exposure. For our bile acid assays, the bile acids were already in the media upon inoculation, whereas the antimicrobial peptide studies typically inject them at a certain point of growth. Other studies determined that bacteria exposed to bile acid stress caused drastic changes in cell morphology, having a shrunken appearance and displaying leakage. It could be hypothesized that the decrease in ZP here should be interpreted as a shrunken membrane that has been leaking [62]. Other studies also analyzed the effects of DCA on membrane integrity, but rather use markers such as DiBAC4 (bis-(1,3-dibutylbarbituric acid) trimethine oxonol), where it was also determined that DCA indeed is membrane disruptive in gram-positive bacteria [17]. The proteomic analyses also provide further hints for membrane disruption by the abundance pattern of the mevalonate pathway. This pathway leads to the production of farnesyl diphosphate, which is required for adding lipid anchors to protein-bound proteins and the pathway is downregulated in the co-culture. Bile acids cause genotoxicity and the DNA damage is reported to be caused by reactive oxygen species (ROS) [60]. Thus, our finding of downregulated proteins related to DNA repair in the co-culture seems to confirm that the membrane, indeed, is less permeable and hence leads to less damage to the DNA via ROS or other stressors. Beside the classical pathways for bile acid tolerance, we identified in *A. caccae* at least two more, namely, the terpenoid and the rhamnose pathway. Both became only detectable after in-detail scrutiny of other involved pathways. The strongly affected terpenoid pathway might be affected but the proteins detected in this study are the more generally related to it. Namely, the ACAT acetyl-CoA C-acetyltransferase [EC:2.3.1.9] and the dxs—1-deoxy-D-xylulose-5-phosphate synthase [EC:2.2.1.7]. The more specific ones for the terpenoid backbone synthesis such as ispf 2-C-methyl-D-erythritol 2,4-cyclodiphosphate synthase [EC:4.6.1.12], the FDPS—farnesyl diphosphate synthase [EC:2.5.1.1 2.5.1.10] and the GGPS1—geranylgeranyl diphosphate synthase [EC:2.5.1.1 2.5.1.10 2.5.1.29]. The decreased abundance in the presence in *E. lenta* indicates that the lipid anchored proteins are of specific importance when the bile stress on the membrane is more pronounced and after reduced membrane stress the abundance is downregulated. Another putative resilience mechanism was found within the proteins that led to the detection of the Streptomycin und Carbapenem pathway. Since these antibiotics are known to be synthesized by fungi, we checked the involved proteins identified in this study in more detail. It turned out that three out of the three proteins, namely, inrfbA (glucose-1-phosphate thymidylyltransferase [EC:2.7.7.24]), rfbB (dTDP-glucose 4,6-dehydratase [EC:4.2.1.46]) and rfbC (dTDP-4-dehydrorhamnose 3,5-epimerase [EC:5.1.3.13]), are also part of the biosynthesis of rhamnose, a deoxysugar critical for membrane integrity. The last one, proA (glutamate-5-semialdehyde dehydrogenase [EC:1.2.1.41] that leads to the production of L-proline is also an upregulated metabolite for both CA and DCA stress. When considering the results from untargeted metabolomics, we, in particular, putatively identified upregulated metabolites that were related to nucleotide metabolism, namely inosine and adenine, in particular for *A. caccae* and *C. ramousm*. These finding are somewhat in contrast to what can be observed in the literature. Studies in the gram-positive strain *Ruminococcus bromii* suggests that metabolites related to carbohydrate and nucleotide metabolism to be downregulated during stress with DCA and LCA [17]. However, metabolites were detected in intracellularly and it could, hence, be hypothesized that both are true, although strains and methodologies are different so such comparisons should be undertaken cautiously. 12-epideoxycholic acid is a metabolite of DCA as a consequence of sequential oxidation and epimerization via 12α and 12β HSDH, and this metabolite was upregulated in *A. caccae* co-cultures. Additionally, we do see a high formation of isoDCA from the measurements of oxidized and epimerized bile acids, but we could not say which isoform it was. It could be hypothesized that with 12-epideoxycholic acid being an upregulated metabolite, and given that we do detect isoDCA in *A. caccae* co-cultures, that this isoform indeed is 12-epideoxycholic acid. Since this metabolite is only significantly detected in *A. caccae* it could be expected that some combination of 12α and 12β HSDH is present in this co-culture. Both *A. caccae* and *E. lenta* carry high scoring homologues to the recently annotated 12β HSDH, so determining whether one or both carry such an enzyme remains to be studied [59]. The increased levels of inosine and adenine stem from the degradation of adenosine, which was also reduced in co-cultures. The reduced levels of adenosine in co-culture can be interpreted as an indication of less genotoxicity in co-cultures, since these metabolites relates to the “Purine metabolism” pathway. There have also been investigations into how *Clostridiodies difficile* (*C. difficile*) responds to bile acid stress using proteomics and scanning electron microscopy (SEM). These investigations revealed that CA induced changes in different pathways compared to CDCA, DCA and LCA. Furthermore, it was also suggested that pathways related to butyrate fermentation and Stickland fermentation of leucine were affected, as well as changes in morphology, such as loss of flagella [63]. If these observations are considered in the context of our findings, there is some overlap when considering changes in metabolic pathways and how bile acid response is specific to the bile acid. The formation of oxidized and epimerized forms of CA and DCA helps explain the reduced toxicity seen in strains such as *C. ramosum*. What drives bile acid toxicity is their amphiphilic nature and their hydrophobicity, and it has previously been described that iso forms of bile acids are less toxic [25,64]. The formation of 3-ketoDCA could, thereby, help explain the differences seen in both growth and ZP of *C. ramosum*, since our data also suggests that *E. lenta* rather rapidly forms the keto and iso forms. We could detect them after only 6 h of growth, whereas other studies have used 24 or 48 h [32,37,59]. Oxidized and epimerized bile acids are less likely to undergo dehydroxylation [32] and performing these transformations may, thereby, further help bacteria reduce stress from bile acids, both via formation of less toxic species and inhibition of formation or more toxic ones such as DCA and LCA. Specifically, what the influence of oxidized and epimerized bile acids are remains to be elucidated, but it has been suggested that isoDCA increased Foxp3 by acting on dendritic cells to diminish their immunostimulatory properties, with 3-oxoDCA exhibiting similar effects but to a lesser extent [65]. 3-ketoLCA has also been shown to be a potent agonist for the Vitamin D Receptor [66]. It was only recently that MBSCs were discovered and this finding (revolutionized the bile acid community since it) brought light a new mode of action by the gut microbiota, namely re-conjugating bile acids with novel amino acid residues [36]. Unconjugated bile acids are more toxic compared to their conjugated counterparts [22,67]. Hence, the formation of MBSCs could be expected to reduce toxicity. In our study we detect MBSCs in all cultures, see Supplementary Figure S9), where in particular Leu-CA seems to increase when co-cultured with *E. lenta*. However, in our analyses the concentration of MBSCs in the medium supernatant is mostly in the 5–40 nM range even though the starting concentration was 600 µM for CA. With such concentrations of MBSCs, it is not likely to reduce toxicity significantly. It remains to be determined whether *E. lenta* facilitates formation of MBSCs or produces them by itself and exactly by which means bacteria re-conjugate bile acids. The in vivo implications of MBSCs remain to be elucidated, both locally and systematically. 

## 5. Conclusions

In summary, our study reaffirms that a bacterium’s response to bile acid stress depends greatly on both the membrane organization of the bacteria and the hydrophobicity of the bile acid. Co-culturing with *E. lenta* alleviated bile acid stress by modifying bile acids into their oxidized and epimerized forms, which in particular was beneficial for *C. ramosum*. An interesting finding was growth behavior and ZP of the gram-variable strain *A. caccae* did not seem to benefit from co-culturing; however, proteomic and metabolomic investigations did indeed produce pronounced effects. The effects of co-culturing with *E. lenta* is strain specific, as reflected in the differences in proteomic and metabolomic analyses between strains. We found that *E. lenta* is, indeed, a potent bile acid modifier, quickly forming oxidized and epimerized forms of CA and DCA. Surprisingly, all strains from the SIHUMIx model system seem capable of forming the novel MBSCs. Overall, our study highlights the importance of conducting in depth molecular investigations, in order to gain insights that are not immediately apparent. We conclude that the presence of potent bile acid modifiers has a relevant impact on the microbe–microbe interaction and potentially, also, on the microbiome-host interaction. 

## Data Availability

The mass spectrometry proteomics data have been deposited to the ProteomeXchange Consortium via the PRIDE [68] partner repository with the dataset identifier PXD035496. Remaining data can be found in the Supplementary data file (https://zenodo.org/record/7193128#.Y0e9XxrP1aS, accessed on 10 Octorber 2022).

## Supplementary Materials

The following supporting information can be downloaded at: https://www.mdpi.com/article/10.3390/microorganisms10102025/s1. Figure S1: Screening of bile acid concentrations in *B. producta* and *B. theraiotaomicron*; Figure S2: Growth curves of SIHUMIx strains when stressed with cholic acid; Figure S3: Additional analyzed bile acids covered in the biocrates bile acid kit from bile acid stress assays; Figure S4: Examples of zeta potential chromatograms; Figure S5: Quality check of proteomic measurements; Figure S6: Principal component analysis of proteomic data; Figure S7: Principal component analysis of untargeted metabolomics data; Figure S8: Oxidized and epimerized bile acids in SIHUMIx cultures; Figure S9: Microbial bile salt conjugations in SIHUMIx cultures; Table S1: YH-BHI media composition: Table S2: Accession numbers of hydroxysteroid dehydrogenase enzymes.
