# Supplementary material for: Eggerthella lenta DSM 2243 Alleviates Bile Acid Stress Response in Clostridium ramosum and Anaerostipes caccae by Transformation of Bile Acids
Source: Microorganisms. 2022 Oct 13;10(10):2025. doi: 10.3390/microorganisms10102025 (PMC9607062; doi:10.3390/microorganisms10102025)
Supplement: Supplementary file 1 [file microorganisms-10-02025-s001.zip › microorganisms-1863465-supplementary/microorganisms-1863465-Supplementary material.pdf]

microorganisms

Eggerthella lenta DSM 2243 alleviates bile acid stress response in *Clostridium ramosum* and *Anaerostipes caccae* by transformation of bile acids

Kristian Jensen Pedersen [1,†], Sven-Bastiaan Haange [1,†], Kateřina Žížalová [2], Alina Viehof [3], Thomas Clavel [3], Martin Leniček [2], Beatrice Engelmann[1], Lukas Y. Wick [4], Frank G. Schaap [5,6], Nico Jehmlich [1], Ulrike Rolle-Kampczyk[1] and Martin von Bergen [1,7,8*]

1 Helmholtz-Centre for Environmental Research—UFZ GmbH, Department of Molecular Systems Biology, 04318 Leipzig, Germany
2 Institute of Medical Biochemistry and Laboratory Diagnostics, First Faculty of Medicine and General University Hospital in Prague, Charles University, Kateřinská 32, 12108 Prague, Czech Republic
3 Functional Microbiome Research Group, Institute of Medical Microbiology, RWTH University Hospital, 52074 Aachen, Germany
4 Helmholtz-Centre for Environmental Research—UFZ GmbH, Department of Environmental Microbiology, 04318 Leipzig, Germany
5 Department of Surgery, NUTRIM School of Nutrition and Translational Research in Metabolism, Maastricht University, 6229 Maastricht, The Netherlands
6 Department of General, Visceral and Transplant Surgery, University Hospital Aachen, 52074 Aachen, Germany
7 German Centre for Integrative Biodiversity Research (iDiv) Halle-Jena-Leipzig, Puschstraße 4, 04103 Leipzig, Germany
8 Institute of Biochemistry, Faculty of Biosciences, Pharmacy and Psychology, University of Leipzig, 04103 Leipzig, Germany
* Correspondence: martin.vonbergen@ufz.de
† These authors contributed equally to this work.

Supplement table S1: YH-BHI media composition
Supplement table S2: Accession numbers of hydroxysteroid dehydrogenase enzymes

Supplement figure S1: Screening of bile acid concentrations in *B. producta* and *B. thetaiotaomicron.*
Supplement figure S2: Growth curves of SIHUMIx strains when stressed with cholic acid.
Supplement figure S3: Additional analyzed bile acids covered in the biocrates bile acid kit from bile acid stress assays
Supplement figure S4: Examples of zeta potential chromatograms.
Supplement figure S5: Quality check of proteomic measurements.
Supplement figure S6: Principal component analysis of proteomic data.
Supplement figure S7: Principal component analysis of untargeted metabolomics data.
Supplement figure S8: Oxidized and epimerized bile acids in SIHUMIx cultures.
Supplement figure S9: Microbial bile salt conjugations in SIHUMIx cultures.

Supplementary Table S1: YH-BHI media composition

Cultivation of individual SIHUMIx strains was performed in Yeast-Hemin-Brain-Heart-Infusion (BHI) medium under

**Ingredient**	**Quantity [g or mL/L]**	**Supplier**
Brain-Heart-Infusion	37	Roth
L-cysteine hydrochloride	0.5	Biochemica
Resazurin	0.001	MP biomedicals
Vitamin K hemin solution	10	Becton Dickinson
Yeast extract	5	Chemsolute

anaerobic conditions. All ingredients are given in Supplement table S1.

BHI medium was aliquoted into Hungate tubes and closed with a butyl cap. Before autoclaving Hungates were purged with pure N_2 and stored at 4C° until use. Strains were thawed using the Cryobank starter set (Mast Group Ltd).

Supplementary Table S2: Accession numbers of hydroxysteroid dehydrogenase enzymes

Shown is a table containing the confirmed HSDH enzymes that were searched against for homologues in the SIHUMIx strains, using the blastp tool from NCBI.

RefSeq	Uniprot ID	Function	Organism
WP_009306474.1	C8WMP0	3α-HSDH	Eggerthella lenta DSM 2243
WP_015760525.1	ACV55294.1	3β-HSDH	Eggerthella lenta DSM 2243
WP_000483353.1	P0AET8.1	7α-HSDH	Escherichia coli K-12 MG1655
WP_009306643.1	C8WLK7	12α-HSDH	Eggerthella lenta DSM 2243
WP_027099077.1	A0A174SBT9	12β-HDSH	Clostridium paraputrificum

Supplementary Figure S1: Screening of bile acid concentrations in *B. producta* and *B. thetaiotaomicron.*

Two representatives from the SIHMUIx model system were selected and exposed to various concentrations of unconjugated bile acids. *Blautia producta* (*B. producta*) was selected for the gram-positive strains and *Bacteroides thetaiotaomicron* (*B. thetaiotaomicron*) for the gram-negative strains. Strains were exposed to 1.2 mM, 0.5 mM, 0.15 mM, 0.05 mM and 0.01 mM of cholic acid (CA), chenodeoxycholic acid (CDCA), deoxycholic acid (DCA) and lithocholic acid (LCA). For LCA only 0.05 mM and 0.01 mM were selected due to solubility issues. BHI media was supplemented with bile acids before inoculation and gassed with pure N_2 after supplementation. Hungates were inoculated with an OD_{600} = 0.1 and measured hourly for 7h. Due to the high inhibition of growth from CDCA and DCA at both 1.2 and 0.5 mM these concentrations were deemed too high. 0.15 mM of these bile acids indicated slight inhibition. Hence for co-culture experiments, DCA concentration was decided to be 0.2 mM.

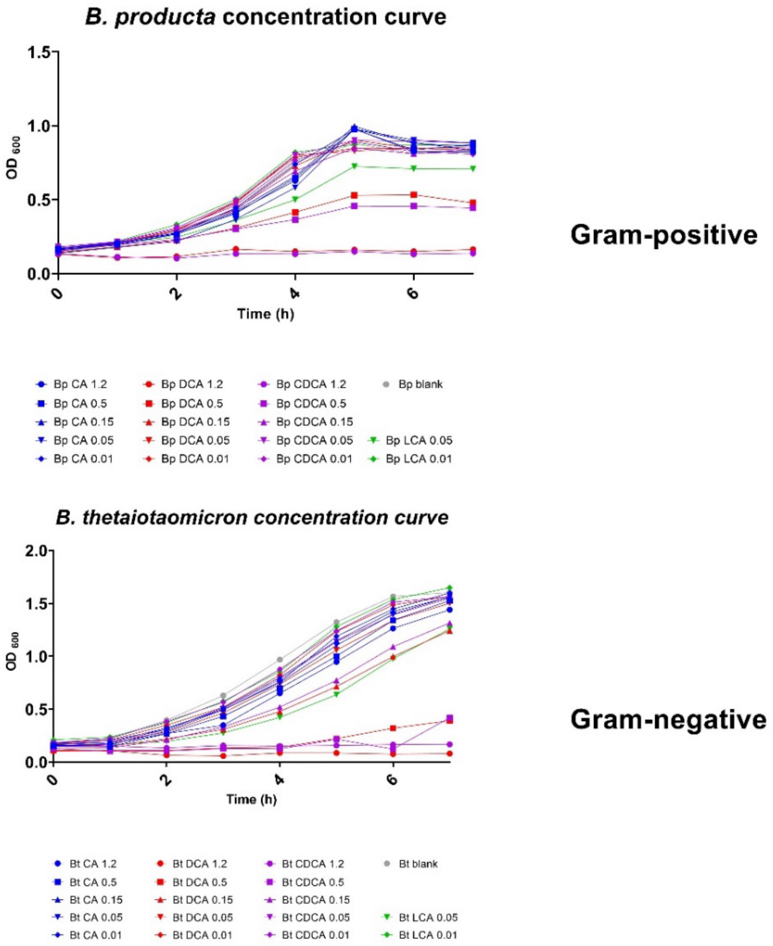

Supplementary Figure S1: Growth curves of *B. producta* and *B. thetaiotaomicron* exposed to bile acids at 1.2, 0.5, 0.15. 0.05 and 0.01 mM. Blue lines are cultures stressed with CA, red lines are cultures stressed with DCA, purple are cultures stressed with CDCA, and green are cultures stressed with LCA. Blanks are represented in grey. Cultures started at an OD_{600} = 0.1 and were measured hourly for 7 hours. Strains were grown in replicates of 1.

Supplementary Figure S2: Growth curves of SIHUMIx strains when stressed with cholic acid.

Growth curves from the bile acid stress experiments are displayed in this figure. The ranking is organized according to inhibition of growth caused by DCA. 600 µM of CA was selected since this is a physiologically relevant concentration encountered in the colon. It can be observed that regardless of strain, CA stress does not seem to affect growth. Differences in final OD_{600} between single and co-culture are suspected to be due to the presence of *E. lenta*.

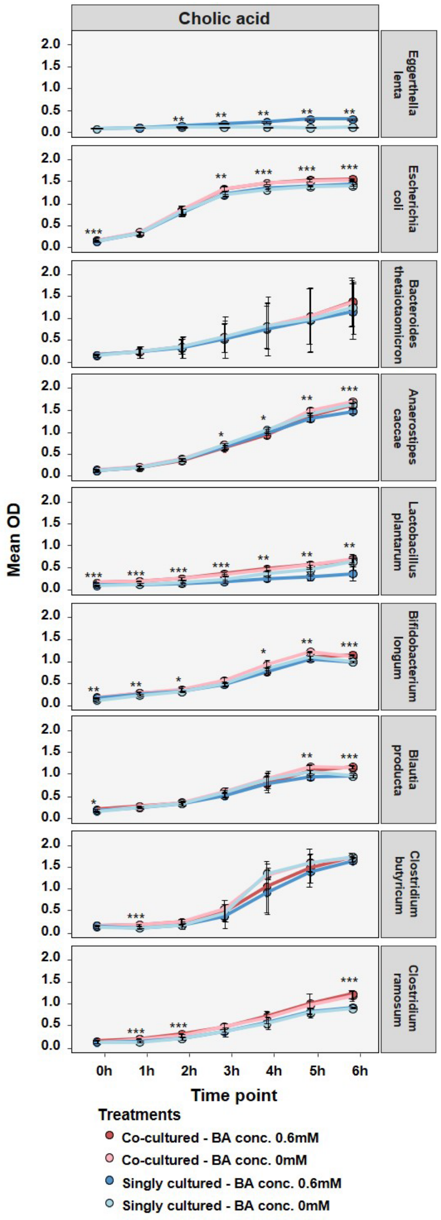

Supplementary Figure S2: Depicted are growth curves of SIHUMIx species in single and co-culture with *E. lenta* with treatment with 600 µM CA. For each experiment, six replicates were analyzed and the line represents the mean. Strains are ranked according to increasing inhibition of growth when stressed with DCA. Error bars are standard deviations. Statistical significance was determined using ANOVA analysis. *p<0.05, **p<0.01, ***p<0.005, ****p<0.001.

Supplementary Figure S3: Additional analyzed bile acids covered in the biocrates bile acid kit from bile acid stress assays.

In addition to determining the decrease in concentration of the initial bile acids, the Biocrates kit also covers other bile acids. An interesting finding is that some murine bile acids seem to be detected when stressed with CA, which should not occur since they are synthesized from CDCA. It could be speculated that this observation is due to a modified bile acid with the same m/z and approximate retention time. Otherwise no other unambiguous trend can be seen.

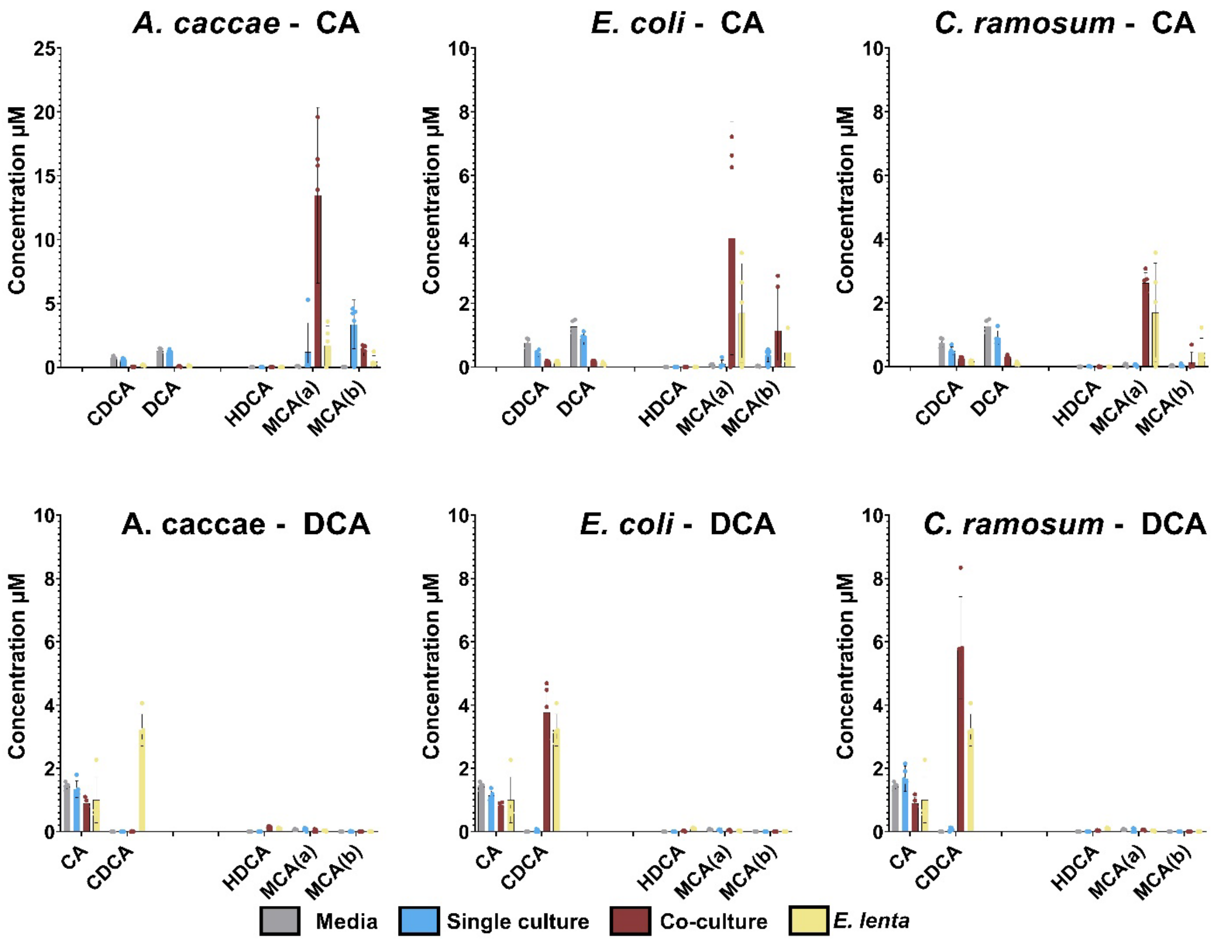

Supplementary Figure S3: Determination of bile acid concentrations in single and co-culture after 6 h of cultivation. For every sample, five replicates were measured and the bars represent the mean value. Grey is the medium, blue is the single culture, red is co-culture and yellow is *E. lenta*.

Supplementary Figure S4: Examples of zeta potential chromatograms

Supplement figure S4 shows averages of chromatrograms obtained by zeta potential measurements. On the left-hand side are single cultures and co-cultures on the right-hand side. Grey lines are blank cultures, blue lines are cultures stressed with CA and red lines are cultures stressed with DCA. Chromatograms were made by extracting data from the ZS XPLORER software into Graphpad Prism. Lines represent an average of 3 chromatograms within each condition. As can be seen, for *E. coli* and *A. caccae* neither bile acid stress nor co-culturing changes the chromatogram. When C. ramosum is observed, it can be seen that DCA shifts the peak towards a more negative zeta potential, where co-culturing brings this peak back towards the blank sample.

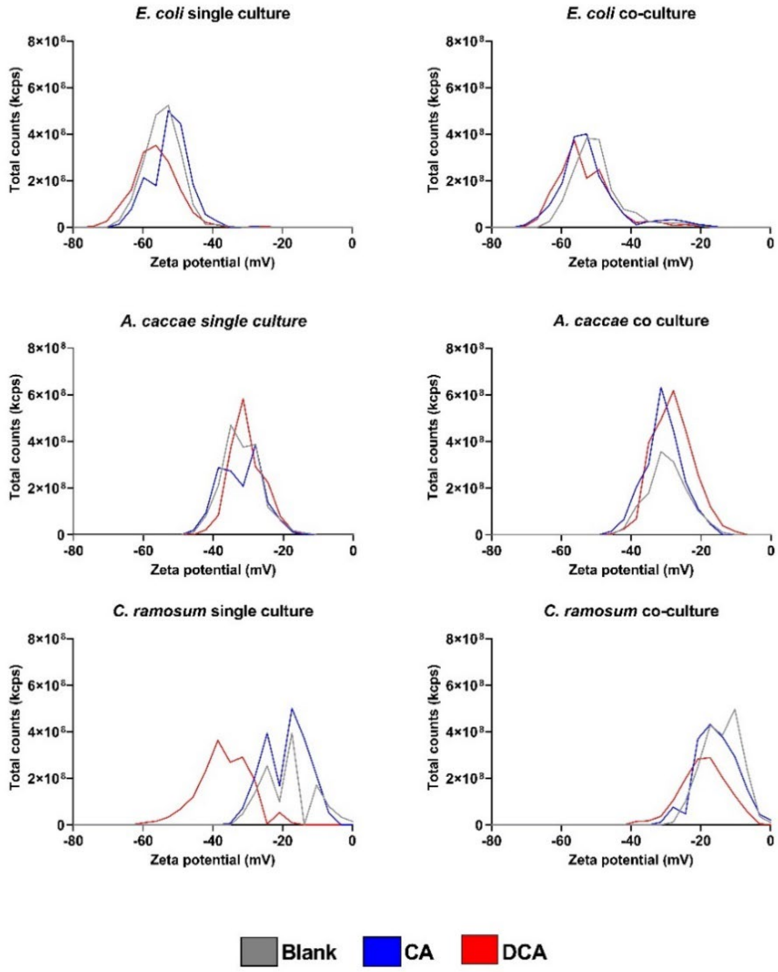

Supplementary Figure S4: Examples of full chromatograms of zeta potential measurements. Left-hand side are single cultures and right-hand side are co-cultures. Grey lines are blank cultures, blue lines are cultures stressed with CA and red lines are cultures stressed with DCA.. 1 mL of washed pellet was used to fill up a capillary cell (DTS1070, Malvern Panalytical Ltd) and measured at room temperature on the Zetasizer Ultra (Malvern Panalytical Ltd) equipped with a He-Ne (633nm) laser. Obtained data were analyzed with the ZS XPLORER (version 2.3.1.4).

Supplementary Figure S5: Quality check of proteomic measurements

Prior to metaproteomic analysis, each strain underwent a quality check before further analyses. From the top row, it can be observed that most protein groups from the samples were assigned to the SIHUMIx strain (red) with a still relevant amount being assignd to *E. lenta* (blue) as well. The bottom row indicates the relative abundances of assigned

protein groups, where it is even more evident that most protein groups from these samples belong to the SIHUMIx strain, but that *E.lenta* is still detectable. This would fit with our observations from the growth curves, that *E. lenta* is not a highly abundant strain.. . For *E. coli* and *A. caccae* it can be observed that both strains are the most dominant ones when co-cultured whereas *C. ramosum* is also but to a lesser extent.

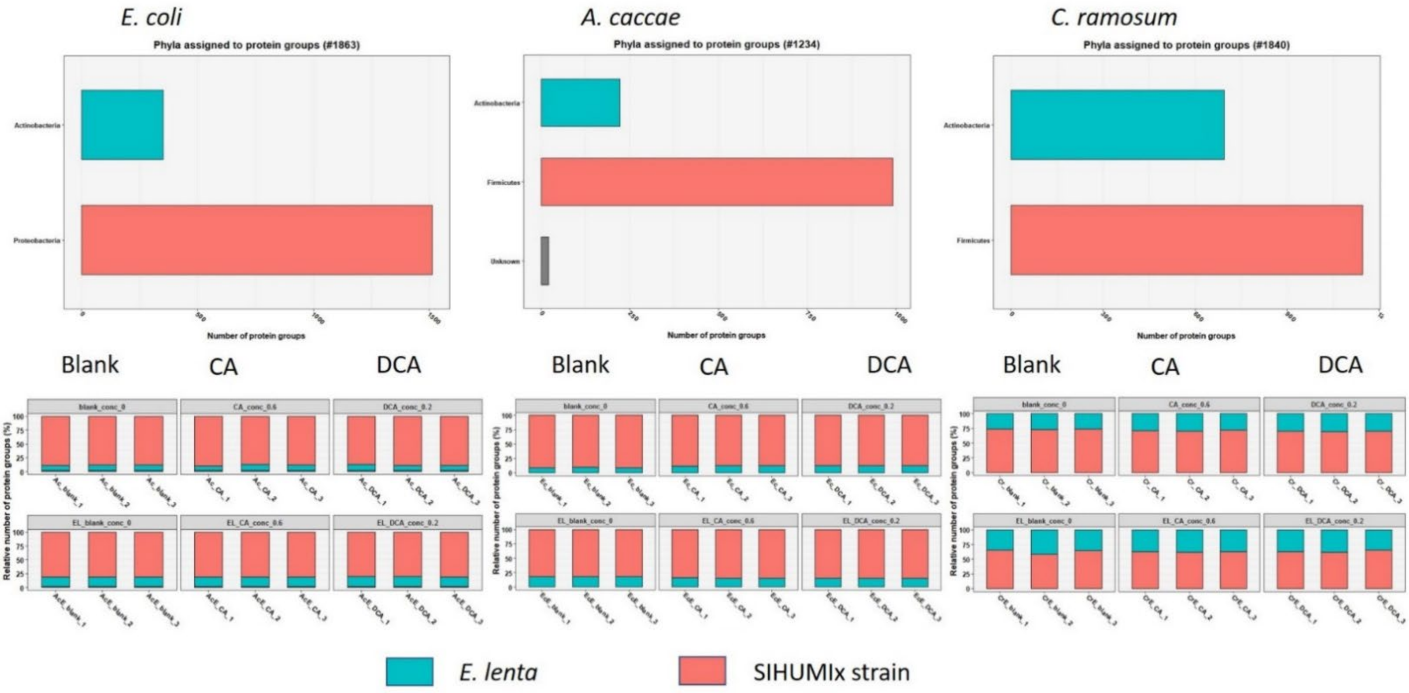

Supplementary Figure S5: The top row indicates protein groups assigned to phyla and the bottom row is the relativeabundances of these protein groups in the various samples. In the relative abundances within samples, the top row represent the single cultures and the bottom part co-cultures. Blue bars represent *E. lenta* and red bars represent a SIHUMIx strain. . Protein intensities were converted to relative abundances by dividing the intensity of the protein by the summed intensities of all proteins from the same species detected in the sample. Protein function assignments were done using Ghostkoala web application from KEGG.

Supplementary Figure S6: Principal component analysis of proteomic data

Principal component analysis (PCA) plots can be seen on supplement figure 6. For *A. caccae* co-culturing in particular drives separation between the groups, in addition DCA also drives this separation within single culture and co-culture. *C. ramosum* does not separate on co-culturing, but rather single culture under DCA stress seems to be a driver of separation. *E. coli* and *E. lenta* no clear separation can be seen under any circumstances.

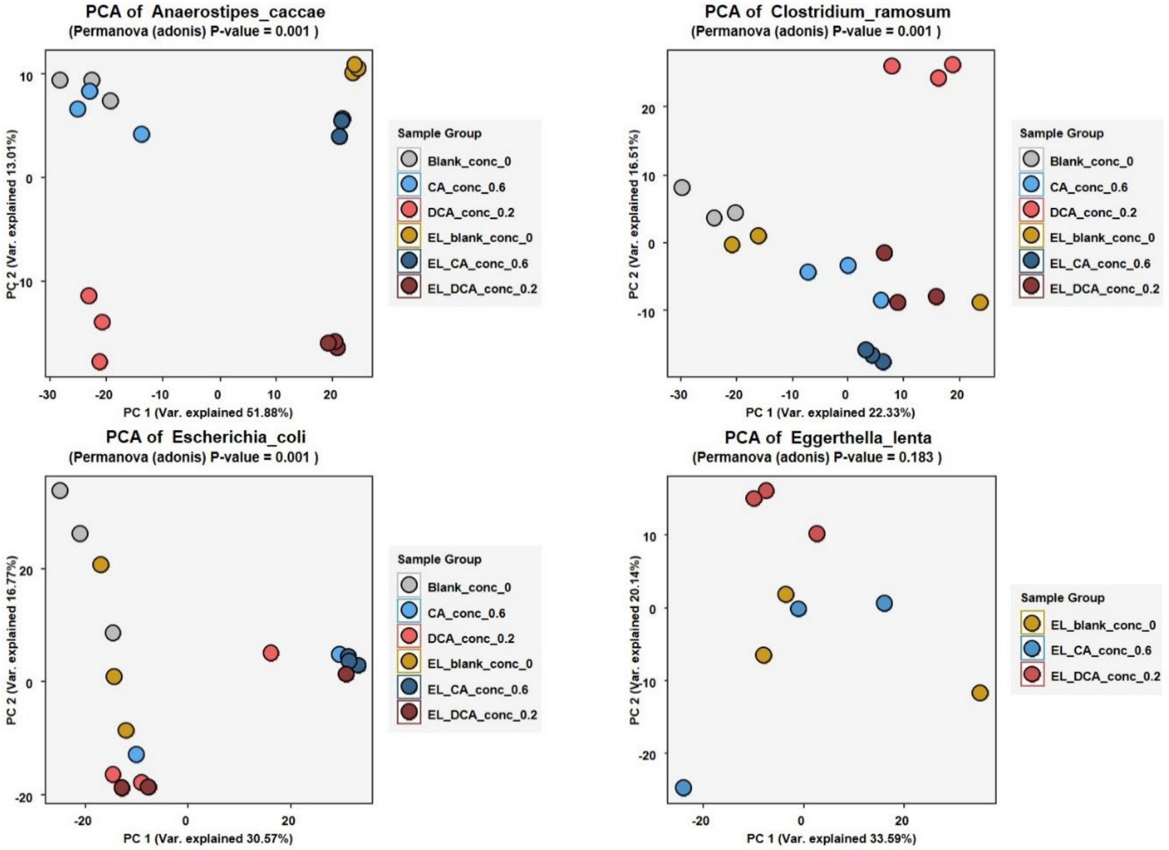

Supplementary Figure S6: PCA plots based on identified proteins harvested from pellets of the bile acid stress experiments.Significance calculated by PERMANOVA using the Adonis function in vegan package for R.

Supplementary Figure S7: Principal component analysis of untargeted metabolomics data

As evident by the PCAS of the untargeted metabolomics, the only trend that is somewhat apperent is the differentiation between single and co-culture. This is true for *E. coli* and *A. caccae* but less obvious for *C. ramosum*

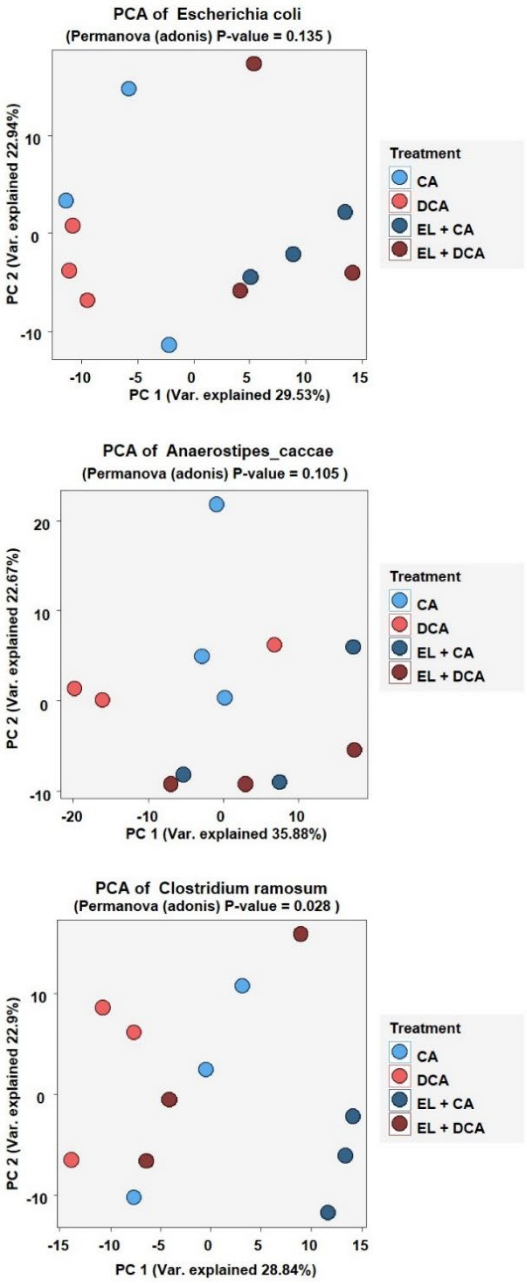

Supplementary Figure S7: Untargeted metabolome analysis of culture supernatants from *E. coli*, *A.caccae* and *C.ramosum*. Bacteria were exposed to CA and DCA as well as co-cultivated with *E. lenta*. Supernatants (n=3) were extracted after 6 hours of cultivation. PCA is based on data of putatively identified metabolites. Differences between treatment groups were calculated with Permanova in R using the adonis function from the VEGAN package.

Supplementary Figure S8: Oxidized and epimerized bile acids in SIHUMIx cultures

The data is semiquantitative since there were no standards available at the time for all the different modifications, hence the data is given as a %, compared to the peak of its original bile acid. For our definition of nomenclature please see Method section "2.8 Measurements of oxo and isoforms of bile acids / MSBCs". *B. thetaiotaomicron* seems to be a potent modifier of CA presumingly converting all CA present in the culture to 7-ketoCA, which is also observed when co-cultured. C. butyricum also seems capable of forming several both iso and keto forms of CA. For DCA however, the trend is clear that only co-cultures express any significant iso or keto form. Blue is single culture, red is co-culture.

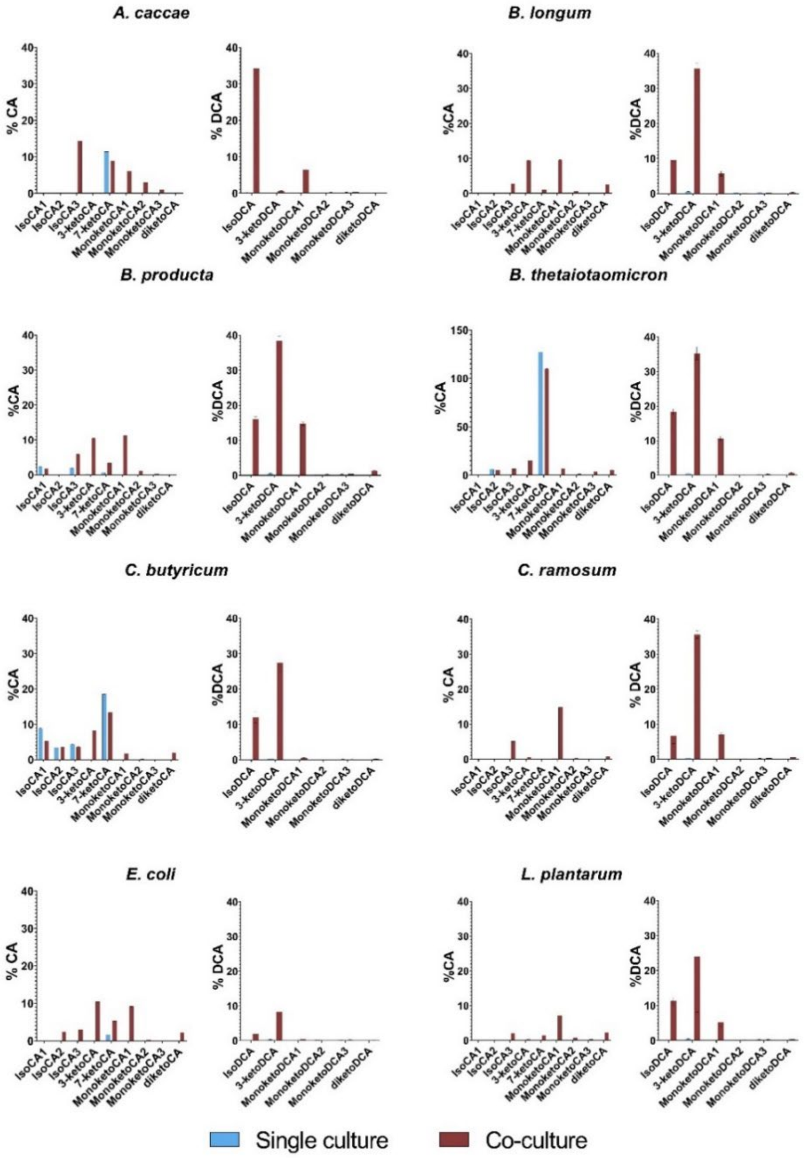

Supplementary Figure S8: Epimerization and oxidization of CA and DCA in single and co-cultures. The bars represent the mean of three biological replicates and the abundances are ratios compared to the peaks of the original added bile acids due to lack of standard for the different iso and keto forms. Blue is single culture and red is co-culture.

Supplement figure S9: MBSC of SIHUMIx strains

Microbial bile salt conjugations (MBSC)s detected in SIHUMIx culutres are depicted on supplement figure S9. Within each strain, left-hand side are cultures stressed with CA and right-hand side are cultures stressed with DCA. Blue is single culture and red is co-culture. It can be observed that all strains seem to have some reconjugation occurring when

stressed with CA in particular. *B. longum*, *B. producta* and *L. plantarum* seem to have a high formation of MBSCs, which is higher than the co-cultured counterpart. When considering DCA stress *C. butyricum*, *C. ramosum* and *L. plantarum* have increased formation when co-cultured.

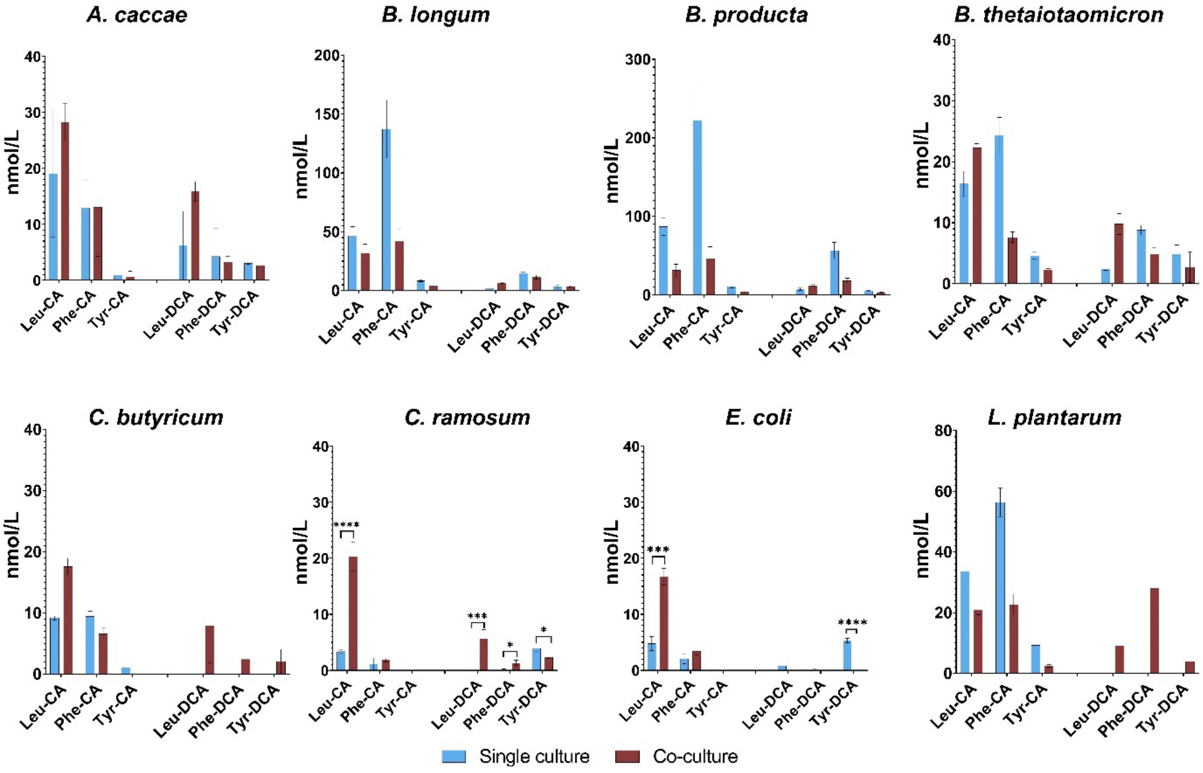

Supplementary Figure S9: MBSCs produced in single and coculture. Bar charts showing the mean concentration from three biological replicates (nmol/L) of the MBSCs estimated from MS measurements of single and co-cultures. Within each strain, left-hand side of the graph are the cultures stressed with CA, and on the right-hand side, those treated with DCA. Blue colors are single cultures and red are co-cultures. Error bars are standard deviations.
